# STF-62247 and pimozide induce autophagy and autophagic cell death in mouse embryonic fibroblasts

**DOI:** 10.1038/s41598-019-56990-y

**Published:** 2020-01-20

**Authors:** Maximilian N. Kinzler, Svenja Zielke, Simon Kardo, Nina Meyer, Donat Kögel, Sjoerd J. L. van Wijk, Simone Fulda

**Affiliations:** 1 Institute for Experimental Cancer Research in Pediatrics, Goethe-University Frankfurt, Komturstr. 3a, 60528 Frankfurt, Germany; 2 Experimental Neurosurgery, Goethe-University Hospital, Theodor-Stern-Kai 7, 60590 Frankfurt, Germany; 3 German Cancer Consortium (DKTK), Partner Site Frankfurt, Frankfurt, Germany; 4 German Cancer Research Centre (DKFZ), Heidelberg, Germany

**Keywords:** Macroautophagy, Macroautophagy

## Abstract

Induction of autophagy can have beneficial effects in several human diseases, e.g. cancer and neurodegenerative diseases (ND). Here, we therefore evaluated the potential of two novel autophagy-inducing compounds, i.e. STF-62247 and pimozide, to stimulate autophagy as well as autophagic cell death (ACD) using mouse embryonic fibroblasts (MEFs) as a cellular model. Importantly, both STF-62247 and pimozide triggered several hallmarks of autophagy in MEFs, i.e. enhanced levels of LC3B-II protein, its accumulation at distinct cytosolic sites and increase of the autophagic flux. Intriguingly, autophagy induction by STF-62247 and pimozide resulted in cell death that was significantly reduced in ATG5- or ATG7-deficient MEFs. Consistent with ACD induction, pharmacological inhibitors of apoptosis, necroptosis or ferroptosis failed to protect MEFs from STF-62247- or pimozide-triggered cell death. Interestingly, at subtoxic concentrations, pimozide stimulated fragmentation of the mitochondrial network, degradation of mitochondrial proteins (i.e. mitofusin-2 and cytochrome c oxidase IV (COXIV)) as well as a decrease of the mitochondrial mass, indicative of autophagic degradation of mitochondria by pimozide. In conclusion, this study provides novel insights into the induction of selective autophagy as well as ACD by STF-62247 and pimozide in MEFs.

## Introduction

The canonical formation of autophagosomes during macroautophagy (hereafter referred to as autophagy) necessarily involves the stages of initiation, nucleation, elongation and closure of the autophagosomal double membrane, followed by recycling and degradation of its contents^1^. Mechanistically, upon the induction of canonical autophagy, the UNC-51-like-kinase 1 (ULK1) complex and the class III phosphatidylinositol 3-kinase (PI3K) complex I initiate the formation of a nascent phagophore on the membrane of the endoplasmic reticulum (ER), which serves as the main donor for autophagosomal membranes^2,3^. The proper formation of the growing autophagosomal membrane requires two ubiquitin-like systems which act in a concerted manner^4^. On the one hand, autophagy-related (ATG)7 and ATG10 covalently attach ATG12 to ATG5 in association with ATG16L1^4^. In parallel, proteins of the Microtubule-associated proteins 1A/1B light chain (LC3)/Gamma-aminobutyric acid receptor-associated protein (GABARAP) family (hereafter referred to as LC3 proteins), which are among the best-studied proteins for monitoring autophagy, are proteolytically cleaved by ATG4B, followed by ATG3- and ATG7-dependent conjugation with phosphatidylethanolamine (PE)^5^. Attachment of PE alters the biochemical properties of LC3 family members to such an extent that these previously cytosolic proteins adopt a membrane-bound state and thus become anchored to the growing autophagosomes^6^. By interacting with a great variety of autophagy receptors, LC3 proteins actively promote the engulfment of bulk cytoplasmic content or selective cellular components such as organelles and protein aggregates. Soon after closure of the autophagosomal membrane, autophagosomes rapidly fuse with lysosomes^7^. The resulting autophagolysosomes finally degrade and recycle the previously engulfed material with the help of lysosomal enzymes such as cathepsins^8^.

Besides bulk autophagy, mitophagy is known as one of the best-characterized forms of selective autophagy. During mitophagy, distinct receptors selectively recognize mitochondria and bridge them to LC3 proteins, which enables lysosomal degradation of the entire organelles^9^. To date, several receptors have been reported to selectively bind to mitochondria, among these are B-cell lymphoma 2 nineteen kilodalton interacting protein 3 (BNIP3), Nix, BCL-2-like protein 13 (BCL2-L-13), FUN14 domain-containing protein 1 (FUNDC1), p62, Optineurin, Calcium-binding and coiled-coil domain-containing protein 2 (NDP52), Protein NBR1 homolog (NBR1) and TAX1-binding protein 1 (TAX1BP1)^10^. A well-characterized model of mitophagy is Phosphatidylinositol 3,4,5-trisphosphate 3-phosphatase and dual-specificity protein phosphatase (PTEN)-induced kinase 1 (PINK1)/Parkin-mediated mitophagy, in which Parkin becomes phosphorylated by the E3 ligase PINK1 and subsequently builds up ubiquitin chains on mitochondria, which are in turn recognized by selective autophagy receptors^11^.

Autophagy is induced in response to starvation, oxidative stress and accumulation of misfolded proteins^12–14^ and can serve to provide novel building blocks in response to high energy demands or as a protein quality control mechanism to remove protein aggregates or damaged organelles^15^. Depending on the cellular context, autophagy may exert dual roles in the regulation of programmed cell death, i.e. lethal and/or cytoprotective. Stimulation of autophagy can promote cell death referred to as ACD^16–19^. Although the molecular signals that trigger ACD are not yet fully understood, there are well-acknowledged criteria to classify cell death as ACD^20^. In particular, genetic knockdown of at least two distinct members of the core autophagic pathway should inhibit ACD^19,20^. In addition, ACD-inducing compounds should enhance the overall autophagic flux instead of blocking the fusion step between autophagosomes and lysosomes^21^. Finally, other types of programmed cell death like apoptosis, necroptosis and ferroptosis should not play substantial roles in the induction of cell death, as ACD represents a type of cell death that is actively promoted by autophagy instead of simply being accompanied by autophagic features as a bystander effect^19^.

On the contrary, autophagy can also serve pro-survival functions. Cytoprotective roles of autophagy have for example been illustrated in several models of neurodegenerative diseases (ND), such as Alzheimer’s disease, Parkinson’s disease, amyotrophic lateral sclerosis (ALS) and Huntington’s disease (HD), where the selective removal of pathological protein aggregates or damaged organelles via selective autophagy can support cell survival^10,22–24^.

Since the induction of autophagy offers novel options to modulate human diseases^25^, the identification and characterization of compounds that stimulate autophagy has attracted special interest. In recent years, several natural compounds, small molecules and Food & Drug Administration (FDA)-approved drugs have been reported to induce autophagy and ACD under different cellular conditions and in different cell types^26–30^. Furthermore, triggering compound-induced autophagy and ACD has attracted particular interest, since many cancer types acquire loss-of-function mutations in conventional cell death pathways, like apoptosis, rendering them resistant against conventional chemotherapies^31^.

Recently, we demonstrated that STF-62247 and pimozide trigger autophagy in glioblastoma (GBM) cells leading to ACD^30^. STF-62247 is an experimental compound that has been shown to elicit autophagy and ACD in renal carcinoma cells^32^ as well as in leukaemia cells^33^. Pimozide is a diphenylbutylpiperidine approved by the FDA for the treatment of schizophrenia that has been described to affect D2 dopaminergic receptors, the 5-HT7 receptor and calcium (Ca^2+^) channels^34,35^. In addition, it has been highlighted by several studies that pimozide induces autophagy in several cellular models^36–38^.

However, at present it remains unclear, if STF-62247 and pimozide can modulate bulk or selective forms of autophagy or even ACD in other prototypic models of autophagy. Therefore, in the present study we explored the efficacy of STF-62247 and pimozide as autophagy-inducing compounds and how these compounds engage lethal autophagy in mouse embryonic fibroblasts (MEFs) as a model system of autophagy.

## Results

### STF-62247 and pimozide trigger autophagy in MEFs

**Figure 1:**
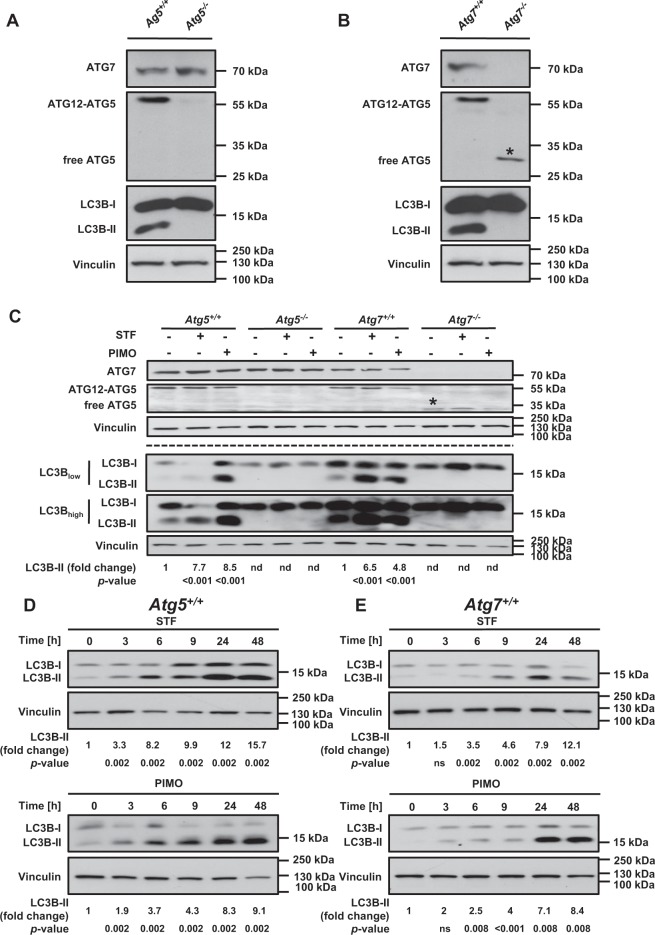
STF-62247 and pimozide trigger autophagy in MEFs. (**A**,**B**) Lysates from untreated *Atg5*^+/+^, *Atg5*^*−/−*^ (**A**) and *Atg7*^+/+^, *Atg7*^*−/−*^ (**B**) MEFs were subjected to Western blotting with vinculin as loading control. The asterisk depicts free ATG5 which is present only in the absence of ATG7. (**C**) *Atg5*^+/+^ and *Atg5*^*−/−*^ MEFs were treated with 20 µM STF-62247 or 15 µM pimozide while *Atg7*^+/+^ and *Atg7*^*−/−*^ MEFs were treated with 40 µM STF-62247 or 10 µM pimozide for 24 hours followed by Western blotting with vinculin as loading control. The asterisk depicts free ATG5 which is present only in the absence of ATG7. The dotted line indicates representations of lysates from two independent experiments. (**D**,**E**) *Atg5*^+/+^ cells were treated with 20 µM STF-62247 or 15 µM pimozide (**D**) and *Atg7*^+/+^ cells were treated with 40 µM STF-62247 or 10 µM pimozide (**E**) for the indicated time points followed by Western blotting with vinculin as loading control. For quantification, LC3B-II protein levels of *Atg5*^+/+^ and *Atg7*^+/+^ MEFs were normalized to vinculin protein levels and expressed as fold changes compared to time point 0. *p*-values were calculated from 3-8 independent experiments. Uncropped blots are presented in Suppl. Fig. 3–7. STF = STF-62247, PIMO = pimozide, ns = not significant, nd = not determined.

In this study, we used MEFs derived from *Atg5* and *Atg7* knockout (KO) mice as well as their corresponding wildtype (WT) counterparts as a cellular non-cancerous model. MEFs are frequently used as model system to study mechanistic aspects of autophagy, and *Atg5*^+/+^*/Atg7*^+/+^ MEFs with the corresponding KO counterparts provide a suitable experimental system with appropriate controls^39–41^. Both ATG5 and ATG7 are key proteins within the building process of autophagosomes^42–45^. As expected, Western blotting confirmed the absence of ATG5 and ATG7 proteins in *Atg5*^*−/−*^ and *Atg7*^*−/−*^ MEFs, respectively (Fig. 1A,B). Besides loss of ATG7 protein expression, *Atg7*^*−/−*^ MEFs did not express the conjugated ATG12-ATG5 protein (Fig. 1B), consistent with the fact that ATG7 is essential for the conjugation of ATG12 and ATG5^18^.

We previously reported that STF-62247 and pimozide increase the autophagic flux of a panel of GBM cell lines^30^. To further evaluate the effects of STF-62247 and pimozide on autophagy induction in MEFs, we initially investigated some of the major hallmarks of autophagy^19,20^. As enhanced lipidation of the autophagosome marker protein LC3B correlates with activated autophagy^20^, we monitored the LC3B lipidation status by immunoblotting. Indeed, treatment with STF-62247 and pimozide stimulated an increase of LC3B-II protein levels in *Atg5*^+/+^ and *Atg7*^+/+^ MEFs compared to untreated cells (Fig. 1C). In sharp contrast, lipidated LC3B-II was completely absent in *Atg5*^*−/−*^ and *Atg7*^*−/−*^ MEFs (Fig. 1C). Additional kinetic analysis revealed that STF-62247 and pimozide induced LC3B lipidation in a time-dependent manner already after three hours of treatment (Fig. 1D,E).

**Figure 2:**
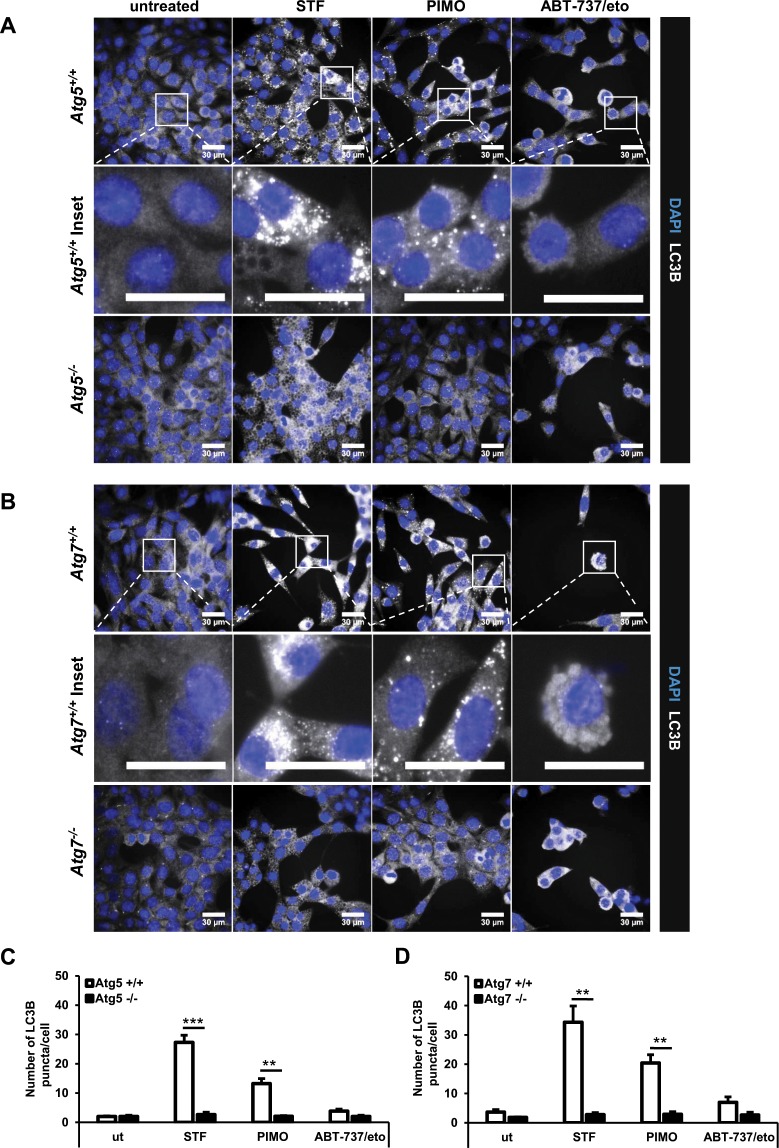
STF-62247 and pimozide lead to a strong accumulation of endogenous LC3B-II protein in MEFs. (**A**,**B**) *Atg5*^+/+^ and *Atg5*^*−/−*^ MEFs were treated with 20 µM STF-62247, 15 µM pimozide or 7.5 µM ABT-737/10 µM etoposide for 24 hours (**A**). *Atg7*^+/+^ and *Atg7*^*−/−*^ MEFs were treated with 40 µM STF-62247, 10 µM pimozide or 7.5 µM ABT-737/10 µM etoposide for 24 hours (**B**). Formation of LC3B puncta was imaged using anti-LC3B immunofluorescence staining. Representative images over 25 sites per sample are shown. (**C**,**D**) Quantification of mean LC3B puncta per cell upon STF-62247, pimozide or ABT-737/etoposide treatment of *Atg5*^+/+^ and *Atg5*^*−/−*^ (**C**) or *Atg7*^+/+^ and *Atg7*^*−/−*^ (**D**) MEFs. Mean and SEM of three independent experiments performed for 25 sites per sample are shown. Significances after drug treatment of *Atg5*^+/+^, *Atg5*^*−/−*^ and *Atg7*^+/+^, *Atg7*^*−/−*^ cells are calculated *versus* untreated cells of the corresponding cell line. Scale bar = 30 µM. **p < 0.01, ***p < 0.001. ut = untreated, STF = STF-62247, PIMO = pimozide, eto = etoposide.

Next, we investigated the localization of endogenous LC3B in STF-62247- and pimozide-treated MEFs, as LC3B is recruited to the growing autophagosomes and can be detected as distinct cytosolic puncta-like structures upon the initiation of autophagy^46,47^. Intriguingly, treatment with STF-62247 and pimozide stimulated pronounced accumulation of endogenous LC3B puncta in *Atg5*^+/+^ and *Atg7*^+/+^ MEFs (Fig. 2A,B). As expected, these were completely absent in ATG5- and ATG7-deficient MEFs (Fig. 2A,B). ABT-737/etoposide-treated cells served as negative control and did not induce LC3B clustering (Fig. 2A,B). Quantification of compound-induced LC3B puncta per cell confirmed that STF-62247 and pimozide substantially increased the number of endogenous LC3B puncta in *Atg5*^+/+^ or *Atg7*^+/+^ cells and that this increase was completely abolished in the corresponding KO cells (Fig. 2C,D). This set of experiments measuring cellular hallmarks of autophagy confirms that STF-62247 and pimozide indeed induce autophagy in MEFs.

### STF-62247 and pimozide enhance the autophagic flux in MEFs

**Figure 3:**
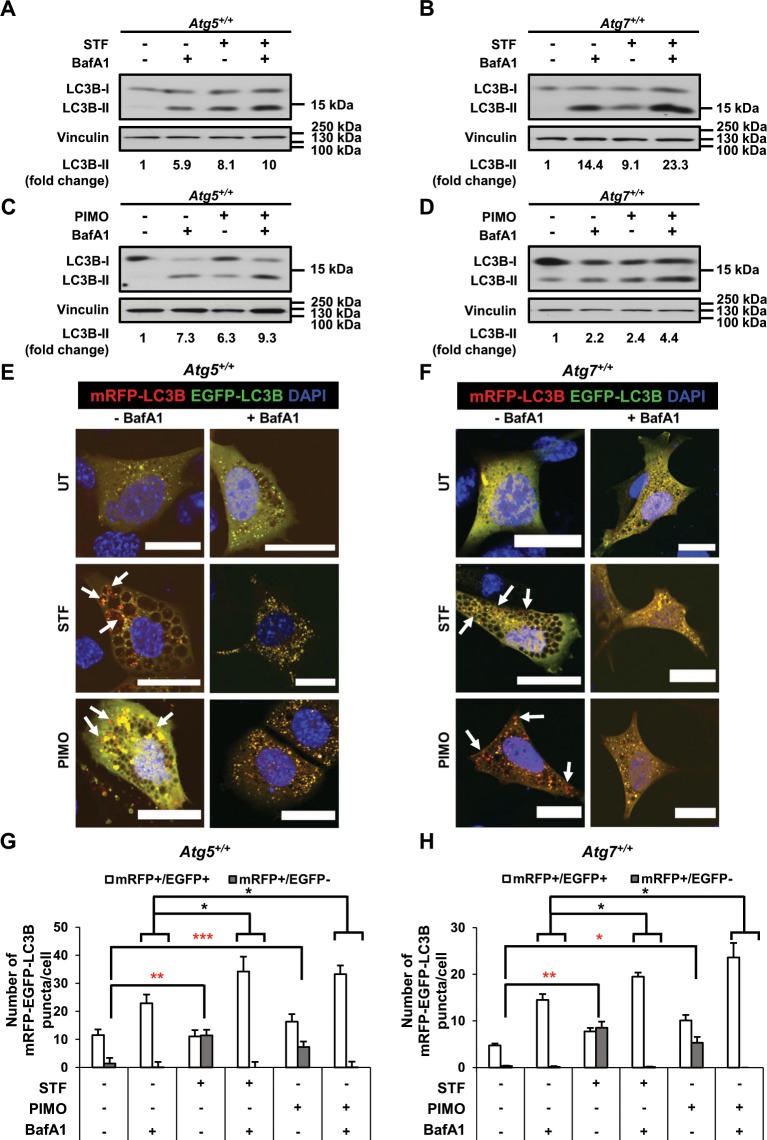
STF-62247 and pimozide lead to enhanced autophagic flux in MEFs. (**A**,**C**) *Atg5*^+/+^ MEFs were treated for 4 hours with 20 µM STF-62247 (**A**) or 15 µM pimozide (**C**) in the absence or presence of 40 nM BafA1. Protein levels were detected by Western blotting with vinculin as loading control. (**B**,**D**) *Atg7*^+/+^ MEFs were treated for 16 hours with 40 µM STF-62247 (**B**) or for 4 hours with 10 µM pimozide (**D**) in the absence or presence of 40 nM BafA1. Protein levels were detected by Western blotting with vinculin as loading control. For quantification, LC3B-II protein levels were normalized to vinculin protein levels and expressed as fold changes compared to the untreated sample. (**E**,**F**) *Atg5*^+/+^ and *Atg7*^+/+^ MEFs were transfected with mRFP-EGFP-LC3B followed by treatment with 20 µM STF-62247 or 15 µM pimozide and 40 µM STF-62247 or 10 µM pimozide in the absence or presence of 40 nM BafA1, respectively, for 8 hours (*Atg5*^+/+^) or for 12 hours (*Atg7*^+/+^). Images were acquired by confocal microscopy. Arrows highlight mRFP^+^ /EGFP^-^ puncta. (**G**,**H**) The numbers of mRFP^+^/EGFP^−^ and mRFP^+^/EGFP^+^ dots per cell were quantified after treatment of *Atg5*^+/+^ (**G**) and *Atg7*^+/+^ (**H**) MEFs with 20 µM STF-62247 or 15 µM pimozide and 40 µM STF-62247 or 10 µM pimozide, respectively, in the absence and presence of 40 nM BafA1. Mean and SEM of three independent experiments are shown. 21-51 cells were quantified per sample. Red stars indicate significances of mRFP^+^ /EGFP^-^ dots in treated *versus* untreated cells. Black stars indicate significances of the sum of mRFP^+^ /EGFP^-^ and mRFP^ +^ /EGFP^+^ dots in cells treated with STF-62247 or PIMO in combination with BafA1 *versus* cells treated with BafA1 alone. Scale bar = 20 µM. Uncropped blots are presented in Suppl. Fig. 8. *p < 0.05, **p < 0.01, ***p < 0.001. ut = untreated, BafA1 = bafilomycin A1, STF = STF-62247, PIMO = pimozide.

Enhanced levels of LC3B-II protein can result either from an increase in autophagic flux or from defective lysosomal degradation^48^. In fact, enhanced autophagic flux is considered as an important prerequisite for the induction of autophagy and is defined as the rate of autophagic degradation activity^20,49^. In order to assess whether STF-62247 and pimozide affect the autophagic flux, *Atg5*^+/+^ and *Atg7*^+/+^ MEFs were treated with STF-62247 or pimozide in the absence and presence of bafilomycin (BafA1), an inhibitor of the lysosomal proton pump V-ATPase^50^. BafA1 has been reported to increase LC3B-II levels by blocking lysosomal degradation of autophagosomes^20,51^. Therefore, an increase in LC3B-II levels in the presence of BafA1 can be interpreted as enhanced autophagic flux^20,51^. Remarkably, we found that the addition of BafA1 to either STF-62247- or pimozide-treated cells caused an increase in LC3B-II protein levels in *Atg5*^+/+^ as well as *Atg7*^+/+^ MEFs in comparison to cells treated with either BafA1, STF-62247 or pimozide alone (Fig. 3A–D). This suggests that both STF-62247 and pimozide indeed enhance the autophagic flux in MEFs.

The effects of STF-62247 and pimozide on autophagic flux were further studied using the well-described tandem fluorescent-tagged LC3B construct that expresses mRFP-EGFP-LC3B fusion protein and can label autophagic compartments at different stages during autophagy^52^. Upon expression of this fusion protein, autophagosomes become apparent as yellow dots, reflecting the colocalisation of mRFP and EGFP^52^. Under lysosomal acidic conditions, EGFP protein loses its fluorescence, whereas mRFP protein remains fluorescent^52^. Thus, upon fusion with lysosomes, the EGFP signal is quenched while mRFP-positive puncta are still present^52^. Intriguingly, the use of this tandem fluorescent-tagged LC3B fusion construct revealed that both STF-62247 and pimozide caused a strong accumulation of mRFP^+^/EGFP^– ^puncta in *Atg5*^+/+^ and *Atg7*^+/+^ MEFs, consistent with an increased autophagic flux (Fig. 3E,F). By comparison, the addition of BafA1 as inhibitor of lysosomal acidification prevented the appearance of mRFP^+^/EGFP^– ^puncta in *Atg5*^+/+^ and *Atg7*^+/+^ MEFs and caused accumulation of yellow-appearing mRFP^+^/EGFP^+^-puncta, consistent with a block in the autophagic flux in the presence of BafA1 (Fig. 3E,F). Quantification of the number of mRFP^+^/EGFP^+^- and mRFP^+^/EGFP^−^-puncta per cell confirmed a marked increase of mRFP^+^/EGFP^−^ puncta upon treatment with either STF-62247 or pimozide in ATG5- or ATG7-proficient MEFs compared to untreated control cells (Fig. 3G,H). This STF-62247- or pimozide-stimulated increase was substantially rescued in the presence of BafA1 (Fig. 3G,H). Moreover, treatment with STF-62247 or pimozide in combination with BafA1 increased the total number of mRFP^+^/EGFP^−^ and mRFP^+^/EGFP^+^ puncta compared to treatment with BafA1 alone (Fig. 3G,H). Together, this set of experiments confirmed that STF-62247 and pimozide enhance the autophagic flux in autophagy-proficient MEFs.

### STF-62247 and pimozide induce autophagy-dependent cell death in MEFs

**Figure 4:**
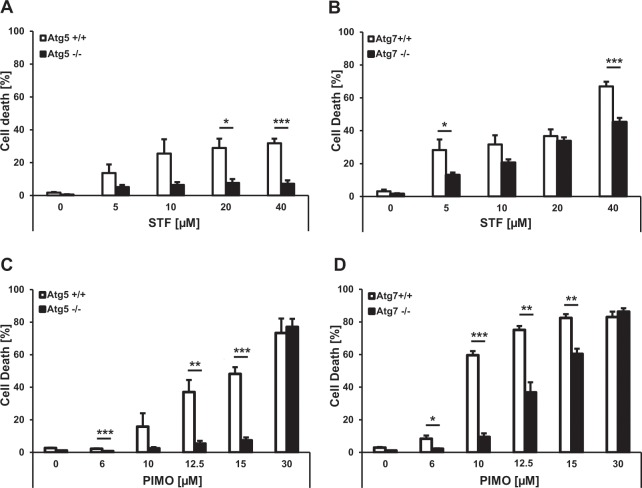
STF-62247 and pimozide induce ATG5- and ATG7-dependent cell death in MEFs. (**A**–**D**) Atg*5*^+/+^*, Atg5*^*−/−*^ (**A**) and *Atg7*^+/+^*, Atg7*^*−/−*^ (**B**) MEFs were treated with the indicated concentrations of STF-62247 for 48 hours. Atg*5*^+/+^*, Atg5*^*−/−*^ (**C**) and *Atg7*^+/+^*, Atg7*^*−/−*^ (**D**) MEFs were treated with the indicated concentrations of pimozide for 48 hours. Cell death was assessed by measuring the PI uptake as fraction of total nuclei determined by Hoechst counterstaining using high-content fluorescence microscopy. Data are presented as mean and SEM of three to five independent experiments performed in triplicate. Significances were calculated against WT cells treated with the same drug concentration. *p < 0.5, **p < 0.01, ***p < 0.001. STF = STF-62247, PIMO = pimozide.

**Figure 5:**
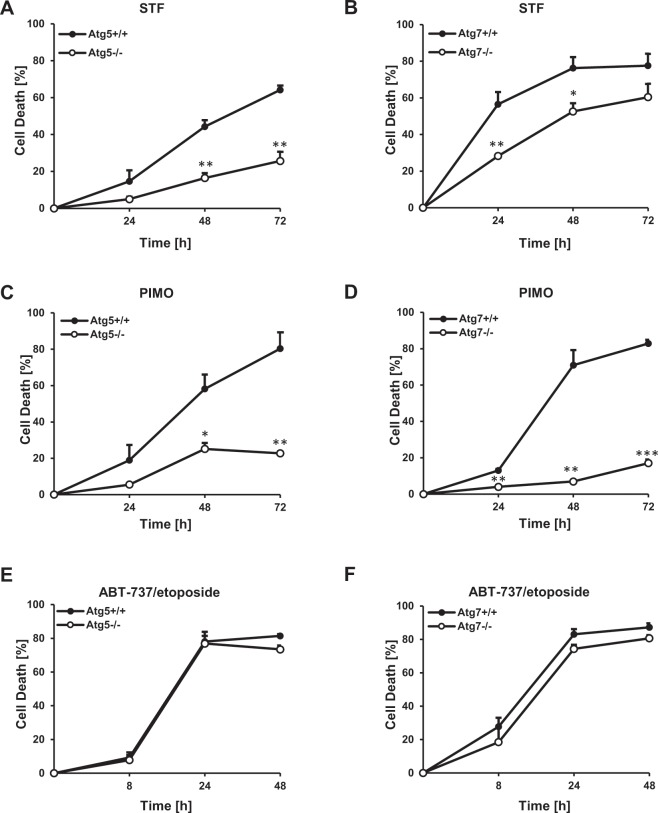
STF-62247 and pimozide induce autophagy-dependent cell death in a time-dependent manner in MEFs. (**A**–**F**) *Atg5*^+/+^ and *Atg5*^*−/−*^ MEFs were treated for 24, 48 and 72 hours with 20 µM STF-62247 (**A**) or 15 µM pimozide (**C**) and for 8, 24 and 48 hours with 7.5 µM ABT-737/10 µM etoposide (**E**). *Atg7*^+/+^ and *Atg7*^*−/−*^ MEFs were treated for 24, 48 and 72 hours with 40 µM STF-62247 (**B**) or 10 µM pimozide (**D**) and for 8, 24 and 48 hours with 7.5 µM ABT-737/10 µM etoposide (**F**). Cell death was assessed by measuring the PI uptake as fraction of total nuclei determined by Hoechst counterstaining using high-content fluorescence microscopy. Data are presented as mean and SEM of three to five independent experiments performed in triplicate. Significances were calculated against WT cells treated with the same drug concentration. *p < 0.5, **p < 0.01, ***p < 0.001. STF = STF-62247, PIMO = pimozide.

It has been speculated that the excessive induction of bulk autophagy beyond a certain threshold can trigger ACD^16,53,54^. Consistently, we previously reported the induction of ACD by STF-62247 and pimozide in GBM cells. To test whether extended autophagy induction by STF-62247 and pimozide induces autophagy-dependent cell death in MEFs as well, ATG5*-* or ATG7*-*deficient MEFs and their corresponding WT counterparts were subjected to increasing concentrations of the compounds, followed by quantification of propidium iodide (PI) uptake. Indeed, both STF-62247 and pimozide triggered cell death in a dose-dependent manner in ATG5- or ATG7-proficient MEFs (Fig. 4A–D). Importantly, this cell death induction was substantially rescued in *Atg5*^*−/−*^ or *Atg7*^*−/−*^ MEFs (Fig. 4A–D), suggesting that STF-62247 and pimozide induce autophagy-dependent cell death in MEFs. For further experiments, we intended to select a drug concentration that would lead to substantial cell killing while at the same time inducing an autophagy-dependent cell death that is clearly reduced in Atg5^*−/−*^ and Atg7^*−/−*^ MEFs. To meet these criteria we selected different concentrations of STF-62247 (20 µM and 40 µM) or pimozide (15 µM or 10 µM) for the *Atg5*^+/+^ and *Atg7*^++−^ MEFs, respectively. A kinetic analysis of cell death revealed that STF-62247- and pimozide-induced cell death increased over time in ATG5- and ATG7-competent MEFs (Fig. 5A–D). Of note, STF-62247- and pimozide-induced cell death was markedly reduced in *Atg5*^*−/−*^ and *Atg7*^*−/−*^ MEFs in a time-dependent manner as well (Fig. 5A–D). In contrast, ATG5 or ATG7 deficiency did not affect ABT-737/etoposide-mediated apoptosis used as a negative control (Fig. 5E,F). Together, these results demonstrate that STF-62247 and pimozide induce ATG5- and ATG7-dependent ACD in MEFs.

### STF-62247- and pimozide-induced cell death shows no major hallmarks of apoptosis, necroptosis or ferroptosis

**Figure 6:**
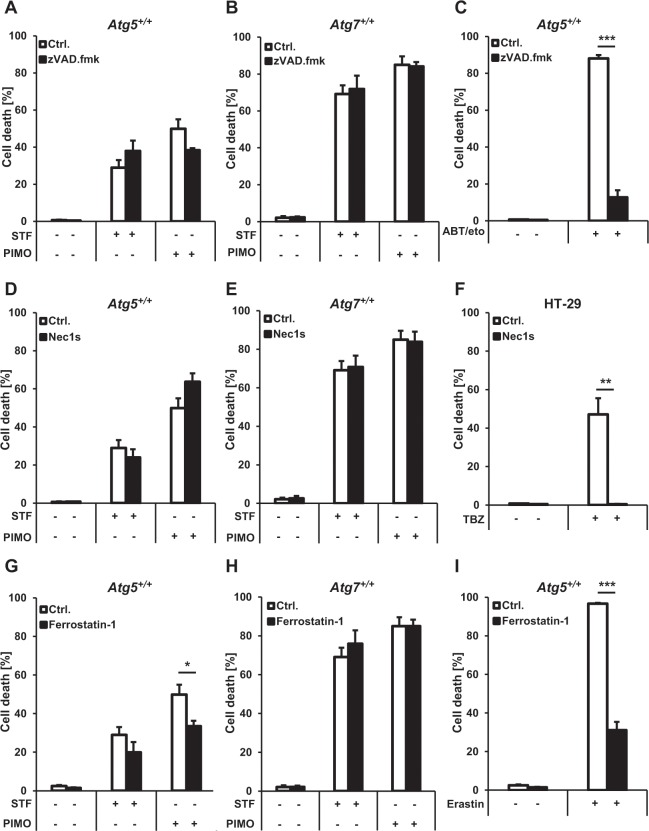
STF-62247- and pimozide-induced cell death shows no major hallmarks of apoptosis, necroptosis or ferroptosis. (**A**,**B**,**D**,**E**,**G**,**H**) *Atg5*^+/+^ MEFs were pre-treated for one hour with 20 µM zVAD.fmk (**A**), 20 µM Nec1s (**D**) or 5 µM Fer-1 (**G**) followed by treatment with 20 µM STF-62247 or 15 µM pimozide for 48 hours. *Atg7*^+/+^ MEFs were pre-treated for one hour with 20 µM zVAD.fmk (**B**), 20 µM Nec1s (**E**) or 5 µM Fer-1 (**H**) followed by treatment with 40 µM STF-62247 or 10 µM pimozide for 48 hours. (**C**) *Atg5*^+/+^ MEFs were pre-treated with 20 µM zVAD.fmk for one hour followed by treatment with 7.5 µM ABT-737/10 µM etoposide treatment for 48 hours. (**F**) HT-29 cells were pre-treated with 20 µM Nec1s for one hour followed by treatment with 1 ng/ml TNFα, 0.5 µM BV6 and 20 µM zVAD.fmk for 48 hours. (**I**) *Atg5*^+/+^ MEFs were pre-treated with 5 µM Fer-1 for one hour followed by treatment with 3 µM Erastin for 48 hours. Cell death was assessed by measuring the PI uptake as fraction of total nuclei determined by Hoechst counterstaining using high-content fluorescence microscopy. Data are presented as mean and SEM of three independent experiments performed in triplicate. Significances were calculated against control cells treated with the same concentration of STF-62247 or pimozide. *p < 0.5, **p < 0.01, ***p < 0.001. Ctrl. = control, STF = STF-62247, PIMO = pimozide, eto = etoposide, TBZ = TNFα, BV6 and zVAD.fmk.

To further strengthen the role of autophagy during STF-62247- and pimozide-induced cell death, we tested whether other modes of programmed cell death such as apoptosis, necroptosis or ferroptosis are required for the induction of cell death by STF-62247 or pimozide. To this end, we measured cell death upon treatment with STF-62247 or pimozide in the absence and presence of pharmacological inhibitors of apoptosis, necroptosis and ferroptosis. Intriguingly, the pan-caspase inhibitor zVAD.fmk failed to block STF-62247- and pimozide-induced cell death in *Atg5*^+/+^ and *Atg7*^+/+^ MEFs (Fig. 6A,B), while it potently suppressed ABT-737/etoposide-mediated cell death that was used as a positive control for caspase-dependent apoptosis (Fig. 6C)^55,56^. Similarly, the Receptor-interacting protein kinase 1 (RIPK1)-inhibitor Necrostatin1 (Nec1)^57^ did not rescue STF-62247- or pimozide-induced cell death (Fig. 6D,E), while a control experiment confirmed that Nec1s provided complete protection in a prototypic model of necroptosis (Fig. 6F), using treatment with the combination of tumor necrosis factor α (TNFα), the Smac mimetic BV6 and zVAD.fmk (TBZ) in HT-29 colon carcinoma cells^58^. Additionally, Ferrostatin-1 (Fer-1), an inhibitor of ferroptosis^59^, largely failed to prevent STF-62247- and pimozide-triggered cell death in *Atg5*^+/+^ and *Atg7*^+/+^ MEFs except for a minor rescue of pimozide-induced cell death in *Atg5*^+/+^ MEFs (Fig. 6G,H), whereas Fer-1 substantially reduced Erastin-stimulated cell death used as a positive control for ferroptotic cell death (Fig. 6I). These findings indicate that neither apoptosis nor necroptosis or ferroptosis are primarily involved in STF-62247- or pimozide-triggered cell death, thus highlighting the role of autophagy in the induction of cell death by STF-62247 or pimozide.

### Pimozide-mediated induction of autophagy induces features of mitophagy in mCherry-Parkin-expressing MEFs

**Figure 7:**
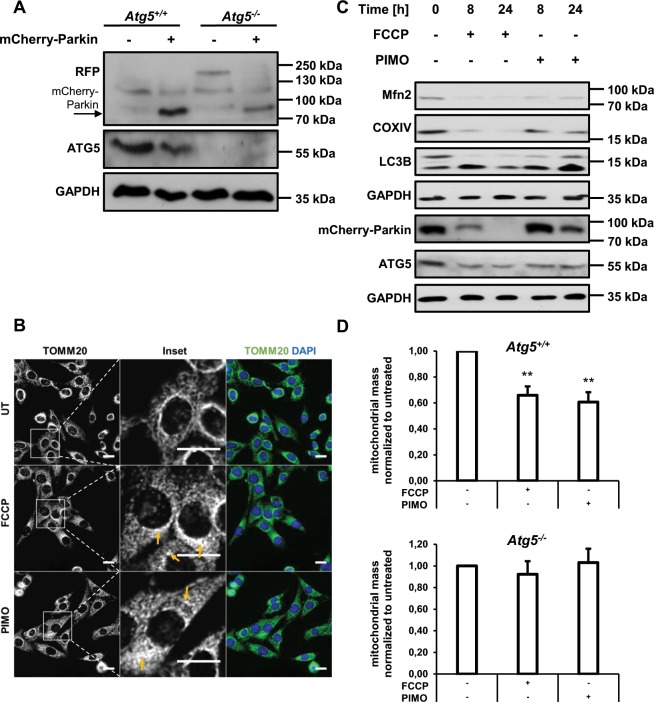
Pimozide-mediated induction of autophagy induces features of mitophagy in mCherry-Parkin-expressing MEFs. (**A**) *Atg5*^+/+^ and *Atg5*^*−/−*^ MEFs were transfected with mCherry-Parkin and expression of mCherry-Parkin was verified by Western blotting with GAPDH as loading control. mCherry-Parkin (expected molecular weight = 81 kDa) is depicted by an arrow. (**B**) mCherry-Parkin-expressing *Atg5*^+/+^ MEFs were treated with 10 µM FCCP or 10 µM pimozide for 8 hours followed by analysis of mitochondrial morphologies using anti-TOMM20 immunofluorescence staining. Arrows depict disruption of the mitochondrial network. (**C**) mCherry-Parkin-expressing *Atg5*^+/+^ MEFs were treated with 10 µM FCCP or 10 µM pimozide for the indicated time points followed by Western blotting with GAPDH as loading control. (**D**) mCherry-Parkin-expressing *Atg5*^+/+^ and *Atg5*^*−/−*^ MEFs were treated with 10 µM FCCP or 10 µM pimozide for 8 hours followed by q-PCR in order to assess the mitochondrial mass determined as the ratio between the DNA levels of the mitochondrial gene *Cytochrome C oxidase* and the nuclear gene *β-actin*. Scale bar = 30 µM. Data are presented as mean and SEM of three to four independent experiments performed in triplicate. Significances were calculated against untreated cells of the same cell line. Uncropped blots are presented in Suppl. Fig. 9–13. **p < 0.01. UT = untreated, FCCP = carbonyl cyanide-4-(trifluoromethoxy)phenylhydrazone, PIMO = pimozide.

Besides the context-dependent engagement of ACD, drugs that induce autophagy have been well-described to trigger selective autophagy, as for example in the context of ND^38,60^. Interestingly, several studies have highlighted a beneficial effect of pimozide treatment on the development of ND, mainly through the induction of selective types of autophagy^36,37,61^. Given the observation that pimozide treatment rapidly induces the autophagic flux in MEFs, we explored the effect of pimozide on modulating one of the best-characterized forms of selective autophagy, i.e. mitophagy. To address this question we used PINK1/Parkin-mediated mitophagy as a well-established model for studying mitophagy and a subtoxic concentration of pimozide. Since MEFs lack endogenous Parkin expression^62^, we stably transfected *Atg5*^+/+^ and *Atg5*^*−/−*^ MEFs with mCherry-Parkin (Fig. 7A). Intriguingly, we noticed that pimozide induced fragmentation of the mitochondrial network (Fig. 7B). The mitochondrial membrane-uncoupling agent carbonyl cyanide-4-(trifluoromethoxy)phenylhydrazone (FCCP) served as positive control (Fig. 7B).

During mitophagy, mitochondria are lysosomally degraded, which eventually results in degradation of mitochondrial proteins and decreased mitochondrial mass^63^. To further investigate whether pimozide triggers degradation of mitochondria, we next assessed protein levels of the outer mitochondrial membrane proteins Mitofusin-2 as well as levels of the inner mitochondrial membrane protein Cytochrome c oxidase subunit 4, isoform A (COXIV). Indeed, pimozide markedly reduced protein levels of Mitofusin-2 and COXIV (Fig. 7C), similar to FCCP. Notably, we observed a similar decrease of Mitofusin-2 and COXIV protein levels upon treatment of mCherry-Parkin-expressing *Atg7*^+/+^ MEFs with pimozide. In contrast, treatment with STF-62247 reduced levels of COXIV protein but not Mitofusin-2 in *Atg5*^+/+^ MEFs, whereas it did not reduce levels of mitochondrial proteins of mCherry-Parkin-expressing *Atg7*^+/+^ MEFs, thus suggesting that STF- 62247 does not trigger mitophagy in MEFs (Suppl. Fig. 1). To confirm that pimozide indeed induces mitophagy, we quantified the mitochondrial mass by determining the DNA ratio between the mitochondrial encoded gene *cytochrome C oxidase* and the nuclear encoded gene *β-actin*^64,65^. Consistent with the obtained Western blot data (Fig. 7C), pimozide markedly decreased the mitochondrial mass (Fig. 7D). This decrease was dependent on ATG5 (Fig. 7D), in line with the notion that PINK1/Parkin-mediated mitophagy uses the canonical autophagic machinery^41,66^. As treatment with pimozide has been reported to be beneficial for treatment of ND, we asked whether pimozide engages the selective degradation of aggregates, i.e. aggrephagy, as well^36,37,61^. In order to exploit the potential role of pimozide in inducing aggrephagy, we induced accumulation of short-lived proteins in *Atg5*^+/+^ MEFs by treating them with the proteasome inhibitor MG-132 (Suppl. Fig. 2), as this has been reported to induce the formation of cytoplasmic aggregates^67,68^. However, we observed no reduction of MG-132-induced protein aggregates assessed by total ubiquitin levels after adding pimozide, thus suggesting that pimozide does not predominantly engage this selective type of MG-132-induced aggrephagy (Suppl. Fig. 2). Together, these experiments highlight a context-dependent selective effect of pimozide in the degradation of mitochondria by autophagy.

## Discussion

In this study, we used MEFs proficient or deficient in one of the key autophagy genes *Atg5* or *Atg7* as a well-established non-cancerous genetic model to study autophagy.

Here, we report that the compounds STF-62247 and pimozide trigger bulk autophagy in MEFs. This conclusion is supported by our findings showing that STF-62247 and pimozide induced several hallmarks of autophagy in ATG5*-* or ATG7*-*proficient MEFs including lipidation of LC3B and accumulation of lipidated LC3B-II at distinct cytoplasmic sites. In addition, both STF-62247 and pimozide enhanced the autophagic flux as documented by both immunoblotting of LC3B and confocal microscopy of mRFP-EGFP-LC3B-expressing cells. Importantly, this engagement of bulk autophagy by STF-62247 and pimozide resulted in ACD in MEFs, since STF-62247- or pimozide-induced cell death was substantially rescued in ATG5*-* or ATG7*-*deficient MEFs compared to the corresponding WT control cells. Also, pharmacological inhibitors of apoptosis, necroptosis or ferroptosis failed to rescue STF-62247- and pimozide-induced cell death in *Atg5*^+/+^ and *Atg7*^+/+^ MEFs, indicating that these other forms of programmed cell death are not predominantly responsible for mediating cell death upon treatment with STF-62247 or pimozide. Thus, our present study characterizes STF-62247 and pimozide as prototypic inducers of ACD in MEFs. By testing different parameters of *bona fide* ACD according to the well-acknowledged guidelines of autophagy^20^, we demonstrated that *Atg5-* or *Atg7-*deficient MEFs represent a cellular tool to study the role of autophagy in drug-induced programmed cell death. The availability of robust cellular genetic KO models such as MEFs will likely facilitate the elucidation of the molecular mechanisms that direct autophagy towards a lethal outcome. However, studies that address ACD induction in MEFs have so far remained scarce^69^. Whereas two studies reported the induction of ACD upon treatment of MEFs with N-desmethyldauricine or upon radiation^70,71^, other studies performed with MEFs focused on the induction of ACD under basal conditions in Puma^*−/−*^, Bid^*−/−*^ and Bax^*−/−*^Bak^*−/−*^ KO MEFs^69,72^.

Interestingly, apart from engaging massive bulk autophagy leading to ACD, we found that pimozide also stimulated mitophagy in MEFs as documented by (1) disruption of the mitochondrial network, (2) degradation of mitochondrial inner and outer membrane proteins and (3) an ATG5-dependent decrease of the mitochondrial mass, suggesting that pimozide has the potential to induce the selective clearance of mitochondria by mitophagy. So far, pimozide has been reported to reduce the levels of mutant Huntingtin along with an improvement of hyperkinesia in models of HD^36,73,74^. Moreover, pimozide has been shown to reduce aggregation of tau protein as well as memory loss in Alzheimer’s disease models via activation of the Adenosine Monophosphate-Activated Protein Kinase (AMPK)-ULK1 axis^37,61^. Our findings add to these studies a previously unknown role in the selective autophagy of mitochondria by pimozide, which may explain some of the reported beneficial effects of pimozide on the progression of NDs^36,37,60^. In line with this notion, defects in mitophagy have been described in several types of NDs such as Alzheimer’s disease, Parkinson’s disease and HD^10^.

Interestingly, parallel induction of bulk autophagy and mitophagy has been observed in several studies^75–77^. For instance, a study performed in yeast suggested that mitochondria are degraded to a certain degree even during starvation-induced bulk autophagy, which was generally thought to be non-selective^75^. In contrast, bulk autophagy has recently been reported to occur in the absence of mitophagy, suggesting that the degradation of mitochondria by pimozide may not simply be the consequence of a generally enhanced autophagic flux^78,79^. Vice versa, a recent study by An and Harper elegantly demonstrated how compounds used to trigger selective autophagy commonly induce autophagic degradation of bulk cytosolic material as well^76^. Additionally, it has previously been speculated that mitochondrial damage which often precedes mitophagy leads to a net loss of energy that will eventually be detected by a cell’s energy sensors, thus inducing bulk autophagy^77^. Together, these studies emphasize possible connections between bulk autophagy and mitophagy.

As we report here that STF-62247 and pimozide trigger strong autophagy which after prolonged treatment induces cell death in MEFs, the question arises as to how this switch from enhanced autophagy to lethal autophagy may be regulated. In fact, it is still being controversially discussed whether autophagy induces ACD through activation of signaling cascades that are specific to this type of cell death or via threshold effects^17^. Indeed, hyper-activation of autophagy itself leading to an increased amplitude and duration of autophagy has been suggested to trigger ACD in a context-dependent manner, which is consistent with the hypothesis that a certain autophagy threshold is required for ACD induction^16–18,53^. Interestingly, in the present study, this threshold effect could explain the induction of lethal autophagy after treatment with STF-62247 and pimozide for 48 hours or longer. Moreover, we speculate that short-time as well as lower-dose treatment with pimozide triggers autophagy as well as selective degradation of mitochondria, but does not exceed the threshold required for lethal autophagy, as we did not detect a marked induction of cell death in these settings. Interestingly, in contrast to our findings that classify STF-62247 as an inducer of autophagic flux and ACD, STF-62247 has recently been reported to block late stages of autophagy by inducing lysosomal disruption in von Hippel-Lindau (VHL)-deficient renal cell carcinoma cells^80^. This study however emphasized that VHL-competent cells are capable to overcome this block of late stages of autophagy and to maintain high lysosome numbers, which is consistent with our data showing that STF-62247 enhances the autophagic flux of VHL-competent MEFs as well as GBM cell lines^30,80^. From a mechanistic perspective, this could be due to STF-62247-induced inhibition the master regulator of autophagy, mTORC1, which we demonstrated in our previous study in GBM cells^30,81^. Besides affecting the autophagic flux, VHL-deficiency was also reported to promote killing of renal cell carcinoma cells either by treatment with low doses of STF-62247 alone (<1.25 µM) or in combination with radiotherapy, whereas the concentrations of STF-62247 used in the present study were markedly higher (20–40 µM)^82^. Consistently, these studies suggest that STF-62247 applied at low doses induces ACD in VHL-deficient cell lines, whereas higher doses are required to induce this type of cell death in VHL-competent cell lines.

Taken together, STF-62247 and pimozide induce ATG5/ATG7-dependent autophagy and ACD in MEFs, characterized by enhanced LC3B-II protein levels as well as activation of the autophagic flux. Interestingly, together with our recent study demonstrating that STF-62247 and pimozide strongly induce ACD in human GBM cells, our present findings highlight the connection between different aspects of autophagy in cancer and healthy cells of human and murine origin. Our results suggest that pimozide is also able to trigger the degradation of mitochondria, highlighting the potential of autophagy inducers to treat a plethora of severe diseases including cancer and NDs and to potentially enhance other types of selective autophagy. In conclusion, our findings provide novel insights into autophagy and will certainly contribute to the elucidation of the highly context-dependent dual role of autophagy.

## Materials and Methods

### Cell lines and chemicals

WT (*Atg5*^+/+^) and KO (*Atg5*^*−/−*^) MEFs were generously provided by Prof. N. Mizushima (Department of Physiology and Cell Biology, Tokyo Medical and Dental University, Japan). *Atg7*^+/+^ and *Atg7*^*−/−*^ MEFs were kindly provided by Prof. M. Komatsu (Tokyo Metropolitan Institute of Medical Science, Japan). MEFs as well as the human colon carcinoma cell line HT-29 were cultured in DMEM GlutaMAX medium (Life Technologies, Inc., Eggenstein, Germany) supplemented with 10% foetal calf serum (FCS) (Life Technologies, Inc., Eggenstein, Germany) and 1% penicillin/streptomycin (Life Technologies, Inc., Eggenstein, Germany) at 37 °C and 5% CO_2_. Cells were regularly tested for mycoplasma infection. HT-29 cells were authenticated by STR profiling at DSMZ (Sammlung von Mikroorganismen und Zellkulturen GmbH). Pimozide, BafA1, Erastin, Fer-1, MG-132 and FCCP were purchased from Sigma-Aldrich (St. Louis, Missouri, USA). Rapamycin was purchased from Enzo Life Sciences (Lausen, Switzerland). STF-62247 was purchased from Santa Cruz Biotechnology, Inc. (Dallas, Texas, USA), etoposide from TEVA GmbH (Ulm, Germany), ABT-737 from Selleck Chemicals (Houston, Texas, USA) and G418 from Carl Roth (Karlsruhe, Germany). The pan-caspase inhibitor zVAD.fmk was purchased from Bachem (Heidelberg, Germany) and Necrostatin-1s (Nec-1s) from Biomol (Hamburg, Germany).

### Generation of ptfLC3-expressing MEFs and determination of autophagic flux

*Atg*5^+/+^, *Atg7*^+/+^ and corresponding KO MEFs were transfected with the ptfLC3 plasmid (Addgene #21074) using FuGENE® HD according to the manufacturers’ instructions. For determination of autophagic flux, cells were seeded on coverslips into Greiner 12-well plates at 65000 cells/well. Prior to imaging, cells were fixed with 3.7% paraformaldehyde for five minutes followed by permeabilization with 0.1% Triton-X100 diluted in PBS for ten minutes. Coverslips were mounted with ProLong™ Diamond Antifade Mountant with DAPI (Life Technologies, Inc., Eggenstein, Germany) and placed on a glass holder. Images were acquired with the Leica SP8 laser-scanning microscope (Leica) using the 63X objective and Type F Immersion Oil (Leica Microsystems, Wetzlar, Germany). Images shown are representative of experiments carried out at least three times. Image analysis was performed with ImageJ (v1.51t). Counting of red and yellow puncta was performed by two independent persons.

### Generation of mCherry-Parkin-expressing MEFs

*Atg*5^+/+^, *Atg5*^*−/−*^ and *Atg7*^+/+^ MEFs were transfected with the mCherry-Parkin plasmid (Addgene #23956) using FuGENE® HD according to the manufacturers’ instructions. 48 hours after transfection, cells were selected with 1 mg/mL G418 (Geneticin®) (Fisher scientific, Hampton, New Hampshire, USA) to generate stable mCherry-Parkin-expressing cell lines.

### Determination of cell death

Cell death was measured by fluorescence-based microscope analysis of PI uptake using Hoechst 33342 and PI double staining (Sigma-Aldrich, St. Louis, Missouri, USA) using the ImageXpress Micro XLS Widefield High-Content Analysis System and MetaXpress Software according to the manufacturer’s instructions (Molecular Devices Sunnyvale, CA, USA).

### Immunofluorescence analyses

For immunofluorescence staining of LC3B, cells were seeded at 7000 cells/96 well. For immunofluorescence, cells were fixed with 3.7% paraformaldehyde for ten minutes, followed by a washing step with PBS and permeabilization with 0.1% Triton-X diluted in PBS for ten minutes. After washing with PBS, cells were blocked with an antibody dilution buffer (ADB) containing 0.9% NaCl, 10 mM Tris HCl pH 7.5, 5 mM ethylene-diamine-tetra-acetic acid (EDTA) and 1 mg/mL BSA for ten minutes. Cells were incubated with an antibody against LC3B (Thermo Fisher, PA1-46286) or translocase of outer mitochondrial membrane 20 (TOMM20) (Santa Cruz, sc-11415) diluted 1:350 or 1:500, respectively, in ADB for one hour at room temperature. After three washing steps with 0.1% Tween-20 diluted in PBS (PBS-T), cells were incubated with fluorescein (FITC) AffiniPure donkey-anti-rabbit IgG (Jackson Immuno Research Laboratories, Inc.) diluted 1:800 in ADB for 30 minutes. After three washing steps with PBS-T, Hoechst 33342 was added to the cells diluted 1:15000 in PBS followed by image acquisition with the ImageXpress Micro XLS Widefield High-Content Analysis System (Molecular Devices Sunnyvale, CA, USA) by using the 60x objective and the DAPI and FITC filter system for acquisition of Hoechst-stained nuclei and FITC-stained LC3B or TOMM20, respectively. Image analysis was performed with ImageJ 1.51t.

### Western blot analysis

Western blot analysis was performed as described previously using RIPA buffer (50 mM Tris-HCl, pH 8, 1% Triton-X, 0.5% sodium deoxycholate, 150 mM sodium chloride and 2 mM magnesium chloride) supplemented with Pierce Nuclease (Thermo Fisher, Waltham, MA, USA)^83^. The following antibodies were used: monoclonal rabbit anti-ATG7, rabbit anti-ATG5 (Cell signalling, Danvers, Massachusetts, USA), mouse anti-GAPDH (Biotrend, Cologne, Germany), mouse anti-vinculin (Sigma, Germany), rabbit anti-LC3B (Thermo Fisher, Waltham, MA, USA), mouse anti-Mitofusin-2, mouse anti-COXIV (Abcam, Cambridge, UK) and rabbit anti-RFP (ChromoTek, Martinsried, Germany). Goat anti-mouse and goat anti-rabbit conjugated to horseradish peroxidase (Santa Cruz Biotechnology, Santa Cruz, CA, USA) as well as enhanced chemiluminescence (Amersham Biosciences, Freiburg, Germany) were used for detection. Representative blots of at least two independent experiments are shown.

### Determination of the mitochondrial mass by quantitative PCR (q-PCR)

In order to determine the mitochondrial DNA mass of MEFs upon treatment with FCCP or pimozide, we quantified the mitochondrial-encoded gene *Cytochrome C oxidase* relative to the nuclear-encoded gene *β-actin*. The ratio of mitochondrial DNA to nuclear DNA reflects the tissue concentration of mitochondria per cell^84^ and can therefore be used to assess the mitochondrial mass. Total DNA was isolated by using the QIAamp DNA Mini Kit (Qiagen, Hilden, Germany) according to the manufacturers’ instructions. For quantification of mitochondrial and nuclear DNA levels, SYBR green-based quantitative real-time PCR was performed using the SYBR™ Green PCR Master Mix (Thermo Fisher Scientific, Darmstadt, Germany) and the QuantStudio 7 Flex Real-Time PCR System (Applied Biosystems, Thermo Fisher Scientific, Darmstadt, Germany). 12 ng of total DNA were used per sample and the q-PCR was initiated with two minutes at 50 °C, followed by ten minutes at 95 °C. This was followed by 40 cycles of 15 seconds at 95 °C and one minute at 60 °C. Analysis of the melting curves served as control for the specificity of the amplified products. Relative DNA levels of the mitochondrial gene *Cytochrome C oxidase* were calculated compared to the nuclear gene *β-actin* by using the 2^−ΔΔ*C*^_T_-method^85^. The resulting mitochondrial mass of MEFs was normalized to the mitochondrial mass of untreated cells of the same cell line. Four independent experiments in technical triplicates were performed for each gene. All primers were purchased by Eurofins (Hamburg, Germany): Cytochrome C oxidase (mitochondrial target gene), forward: ACTCCTACCACCATCATTTCTCC, reverse: GGCTAGATTTCCGGCTAGAGG; *β*-actin (nuclear target gene), forward: GGAAAAGAGCCTCAGGGCAT, reverse: CTGCCTGACGGCCAGG.

### Statistical analysis

Results are expressed as mean +/− SEM. Statistical analysis was performed with SigmaPlot (v12.5). Statistical significance of two group data was analysed by student’s t-test (two-tailed). If samples did not pass either the Shapiro-Wilk Normality Test or the Equal Variance test, statistical significance was analysed by Mann-Whitney Rank Sum Test. *p*-values were interpreted as follows: *p < 0.05; **p < 0.01; ***p < 0.001.

## Supplementary information

Supplementary Information.

## Data Availability

All data generated or analysed during this study are included in this published article (and its Supplementary Information files).
