# Supplementary material for: STF-62247 and pimozide induce autophagy and autophagic cell death in mouse embryonic fibroblasts
Source: Sci Rep. 2020 Jan 20;10:687. doi: 10.1038/s41598-019-56990-y (PMC6971264; doi:10.1038/s41598-019-56990-y)

STF-62247 and pimozide induce autophagy and autophagic cell death in mouse embryonic fibroblasts

Maximilian N. Kinzler[1,3§], Svenja Zielke[1§], Simon Kardo[1], Nina Meyer[2], Donat Kögel[2], Sjoerd J. L. van Wijk*[1], Simone Fulda #*[1,3,4]

[1]Institute for Experimental Cancer Research in Pediatrics, Goethe-University Frankfurt, Komturstr. 3a, 60528 Frankfurt, Germany

[2]Experimental Neurosurgery, Goethe-University Hospital, Theodor-Stern-Kai 7, 60590 Frankfurt, Germany

[3]German Cancer Consortium (DKTK), Partner Site Frankfurt, Germany

[4]German Cancer Research Center (DKFZ), Heidelberg, Germany

[§] shared first authorship, * shared senior authorship, # corresponding author

Pimozide decreases the levels of the mitochondrial proteins Mfn2 and COXIV in *Atg5*+/+ and *Atg7*+/+ cells . STF-26647 decreases levels of COXIV, but not of Mfn2 in *Atg5*+/+ cells, whereas STF-26647 does not decrease levels of COXIV or Mfn2 in Atg7+/+ cells. ATG5$^{+/+}$ and ATG7$^{+/+}$ MEFs were transiently transfected with mCherry-Parkin followed by treatment with 10 µM FCCP, 10 µM pimozide or 10 µM STF-62247 for the indicated time points followed by Western blotting with vinculin as loading control. Uncropped blots are shown in Suppl. Fig. 13. Mfn2 = mitofusin-2

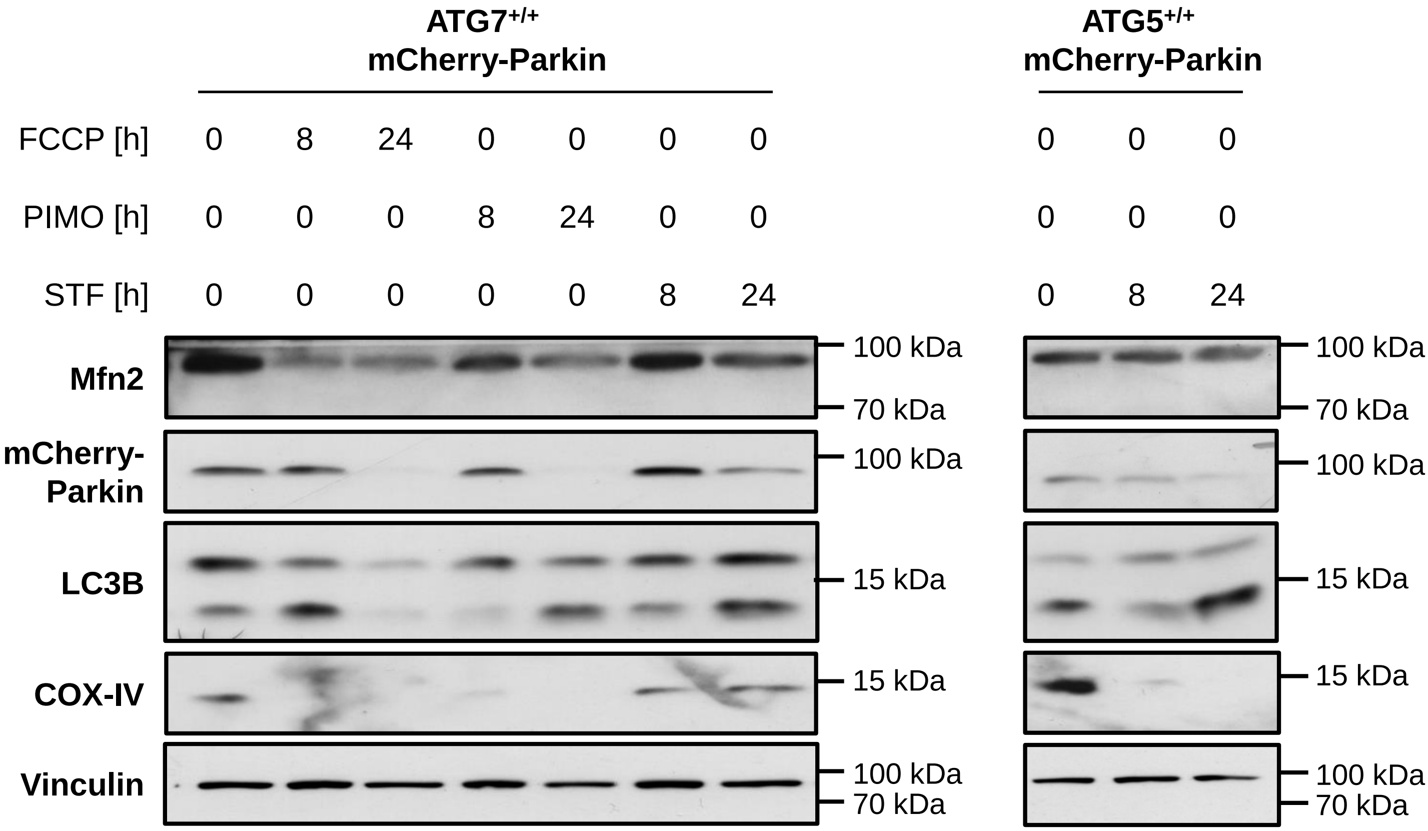

STF-26647 and pimozide do not induce selective autophagy of MG-132-induced aggregates. ATG5+/+ and ATG5-/- MEFs were pre-treated with rapamycin (7.5 uM), STF or PIMO at the indicated concentrations for two hours prior to treatment with MG-132 (3 uM) and the compounds for 18 hours followed by Western Blotting with GAPDH as loading control. Uncropped blots are shown in Suppl. Fig. 14.

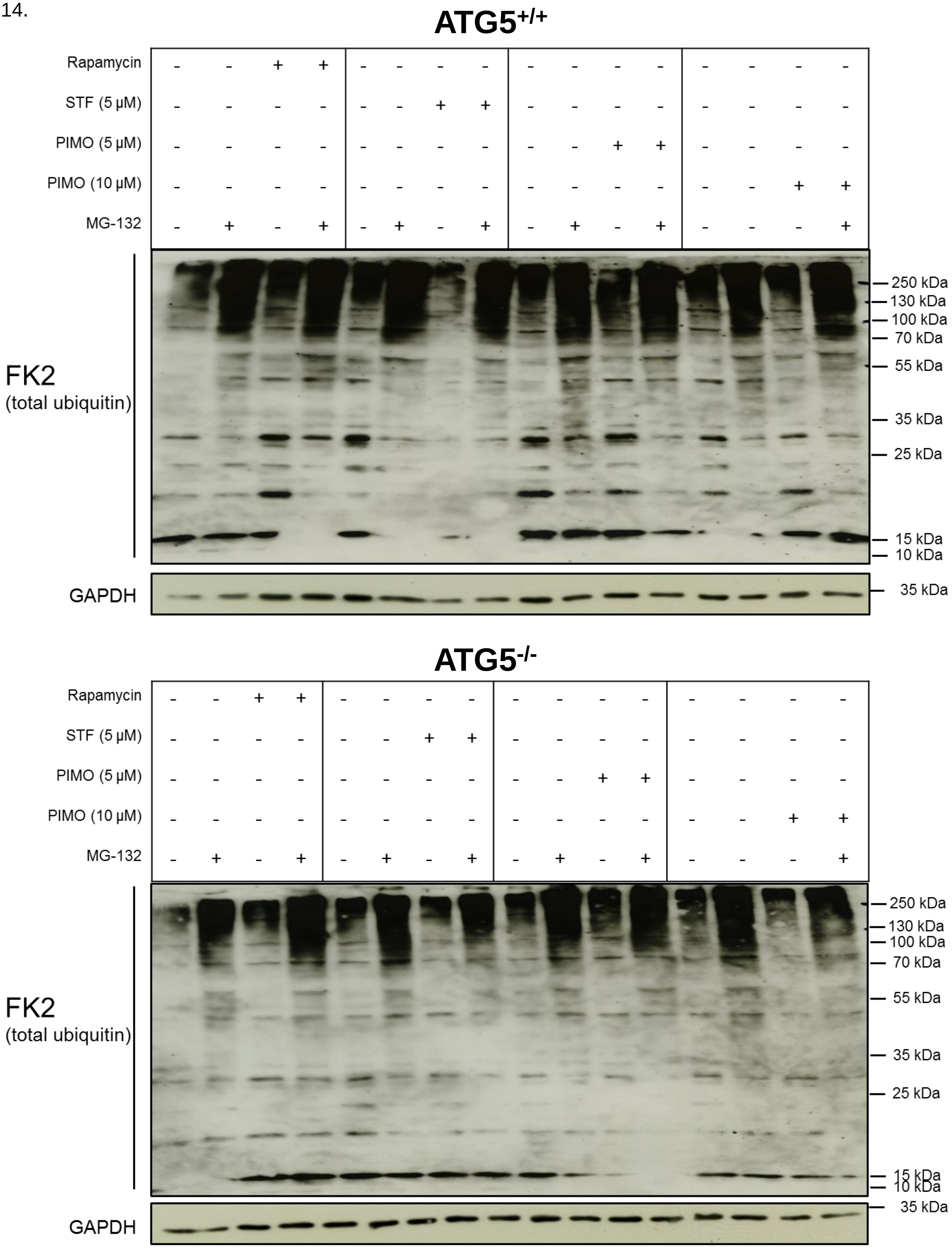

Figure 1A/B: original blots in different exposures

(1) Vinculin, ATG7, LC3B

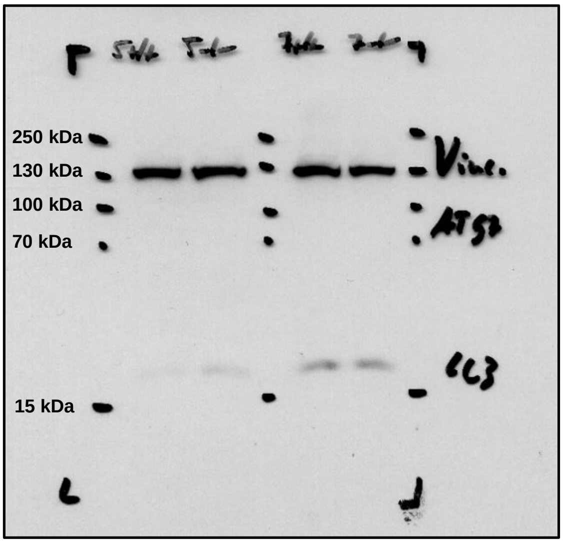

(2) Vinculin, ATG7, LC3B

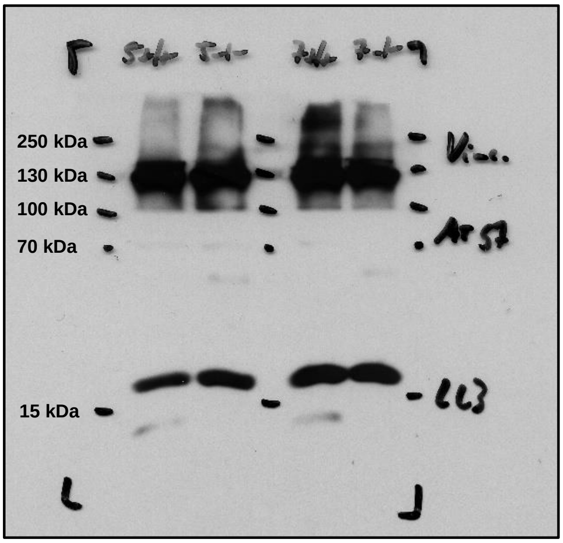

(3) Vinculin, ATG7, LC3B

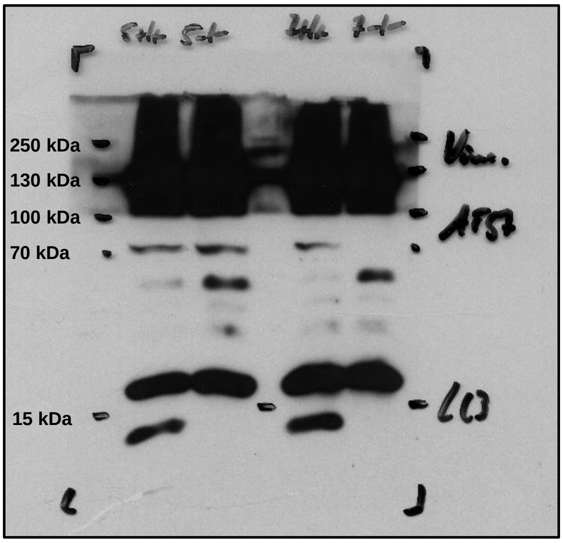

(4) ATG5

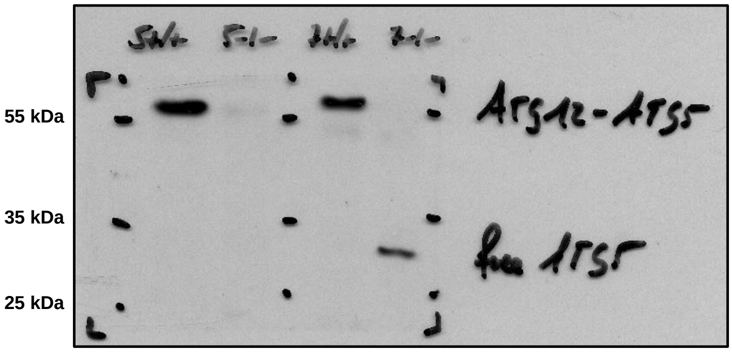

Figure 1C: original blots in different exposures

(1) Vinculin, ATG7, ATG5

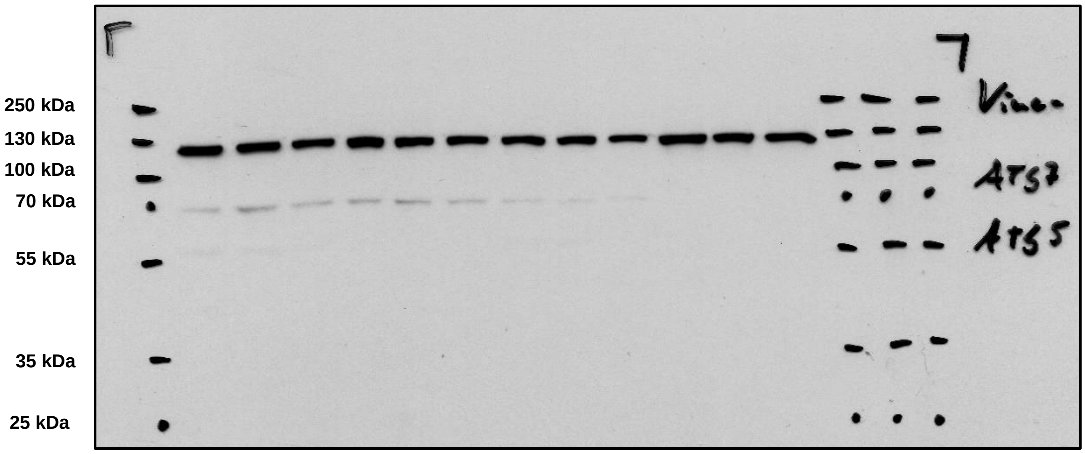

(2) Vinculin, ATG7, ATG5

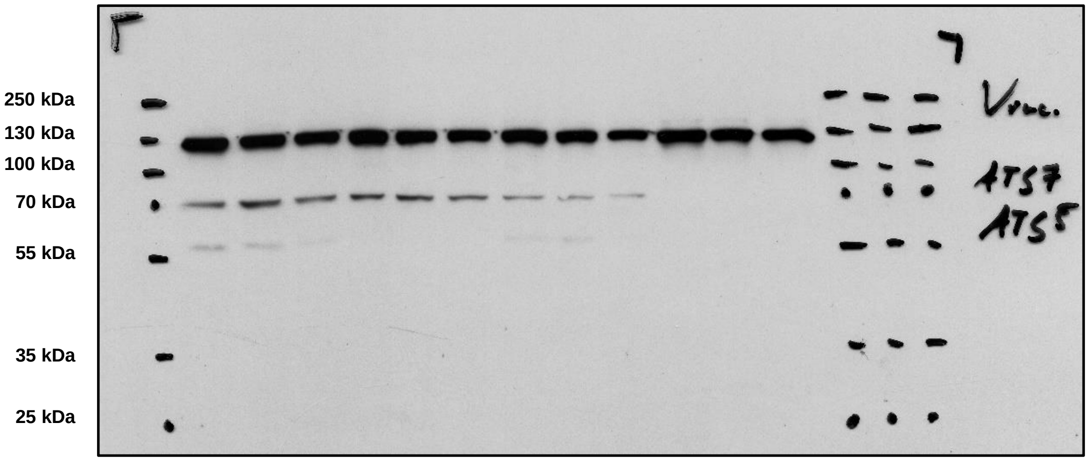

Figure 1C: original blots in different exposures

(3) Vinculin, ATG7, ATG5

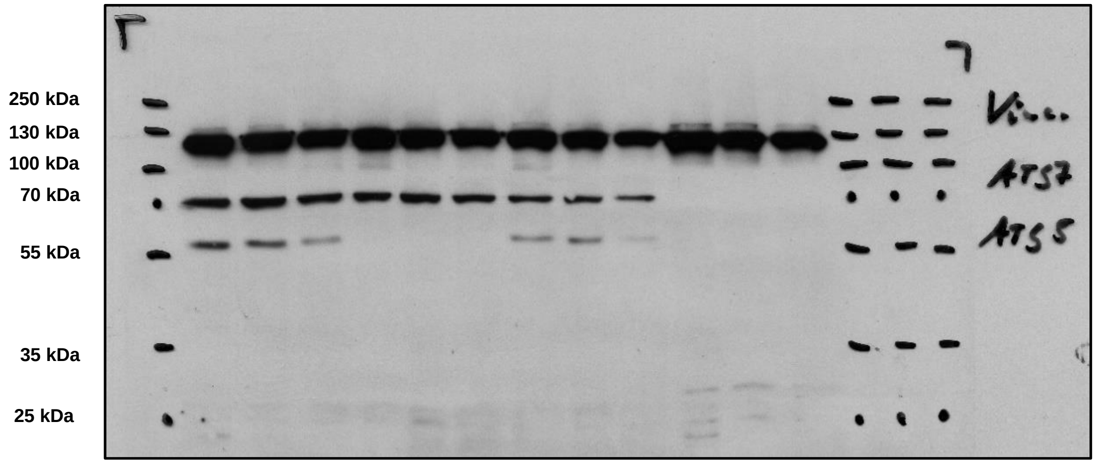

(4) Vinculin, ATG7, ATG5

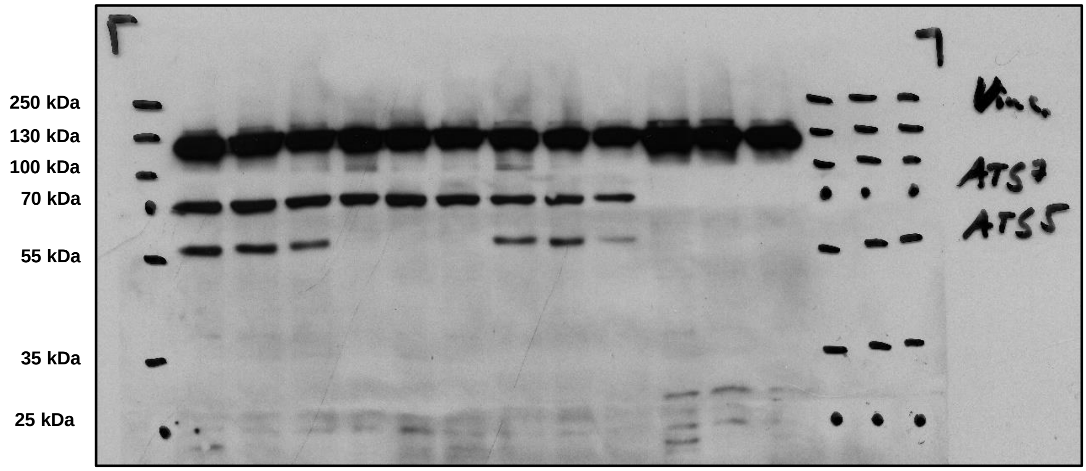

Figure 1C: original blots in different exposures

(5) Vinculin

250 kDa
130 kDa
100 kDa

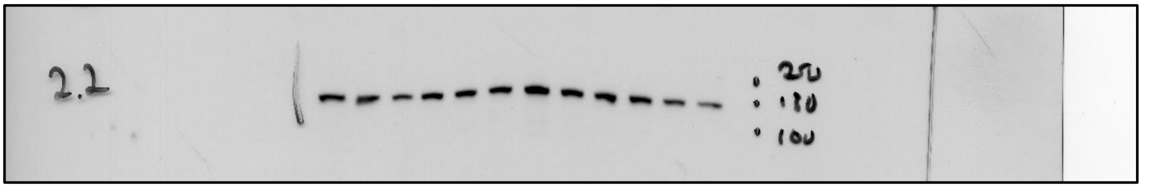

(6) LC3B

15 kDa

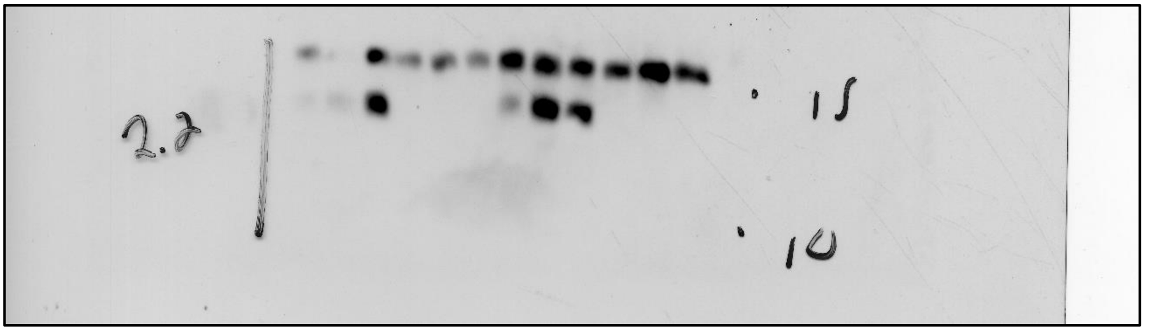

(7) LC3B

15 kDa

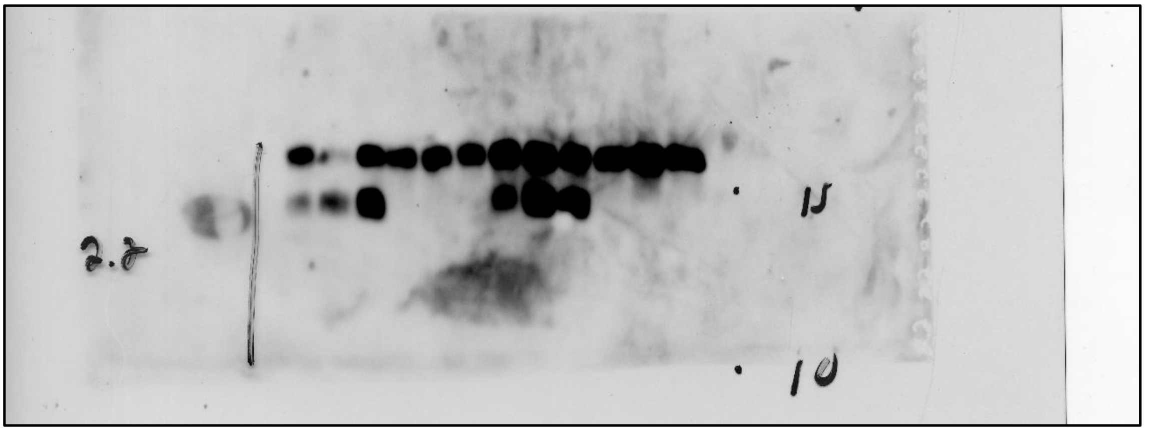

Figure 1D/E: original blots in different exposures

Suppl. Fig. 7

Marked samples are part of the present study.

(1) Vinculin

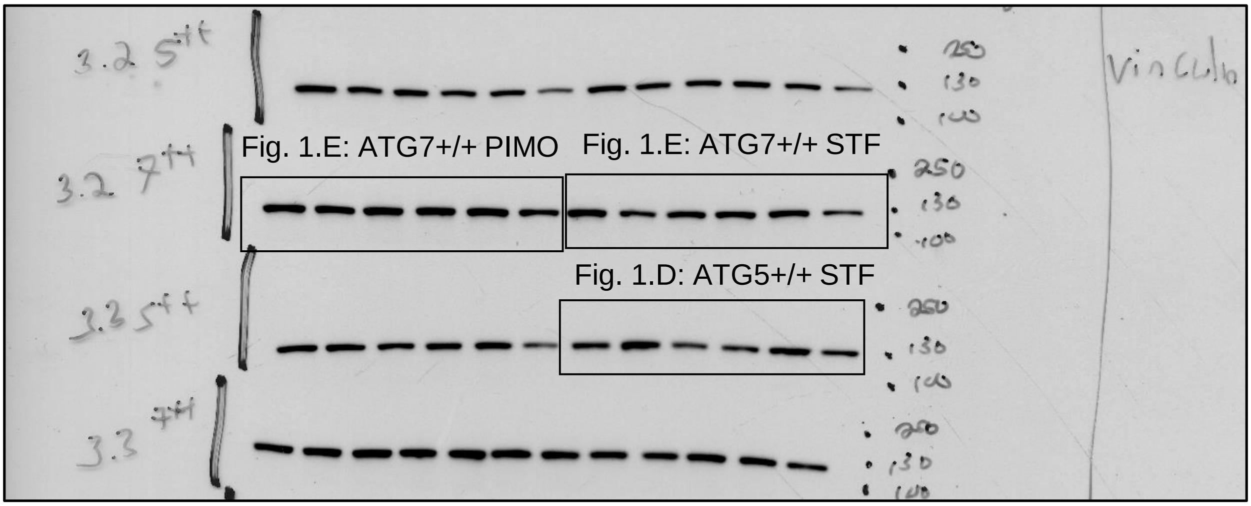

(2) Vinculin

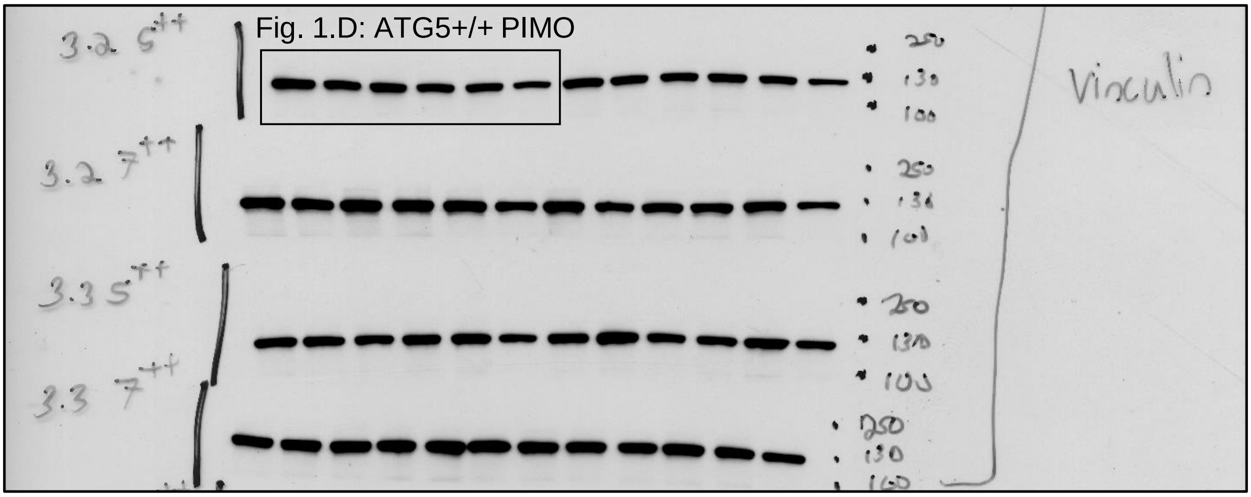

(3) LC3B

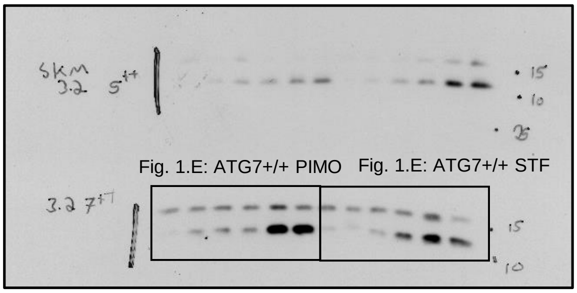

(4) LC3B

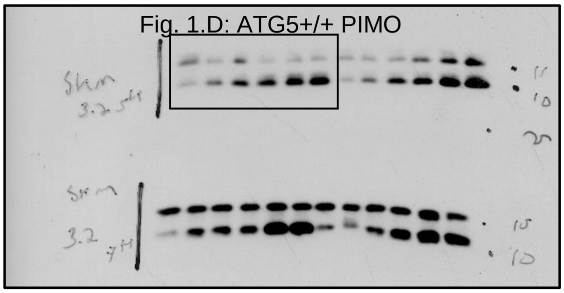

(5) LC3B

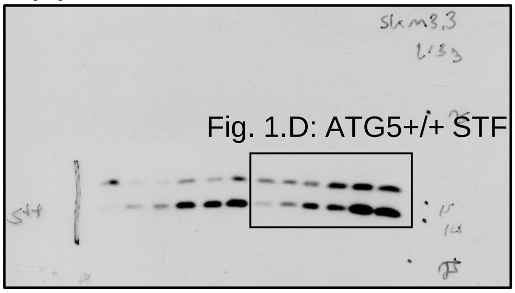

Figure 3A/B/C/D: original blots in different exposures

Suppl. Fig. 8

Marked samples are part of the present study.

(1) Vinculin

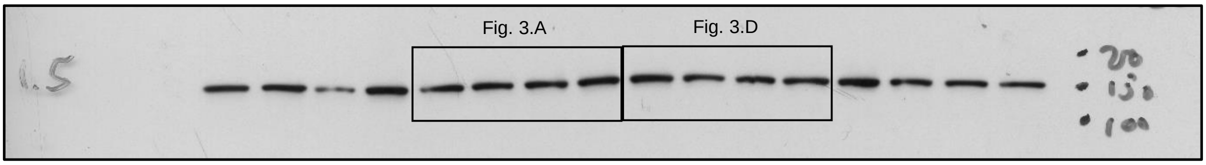

(2) Vinculin

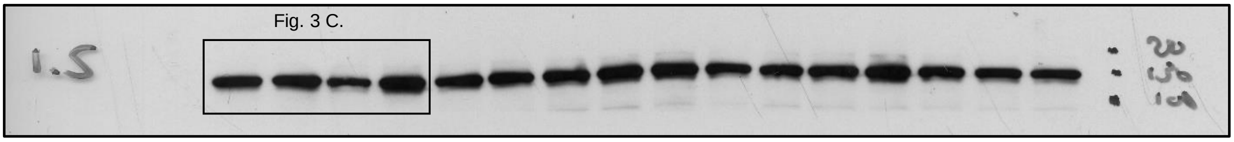

(3) LC3B

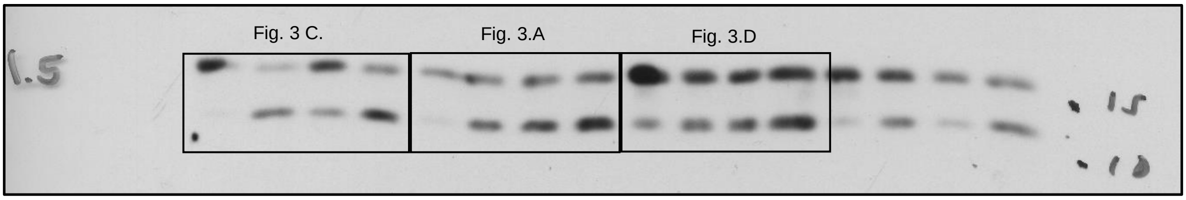

(4) Vinculin

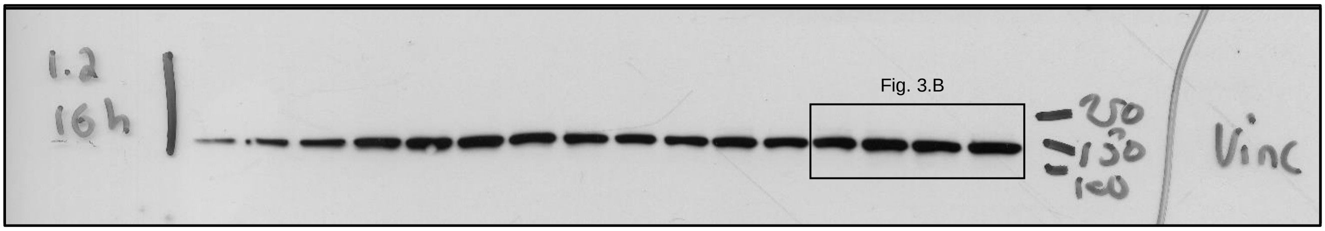

(5) LC3B

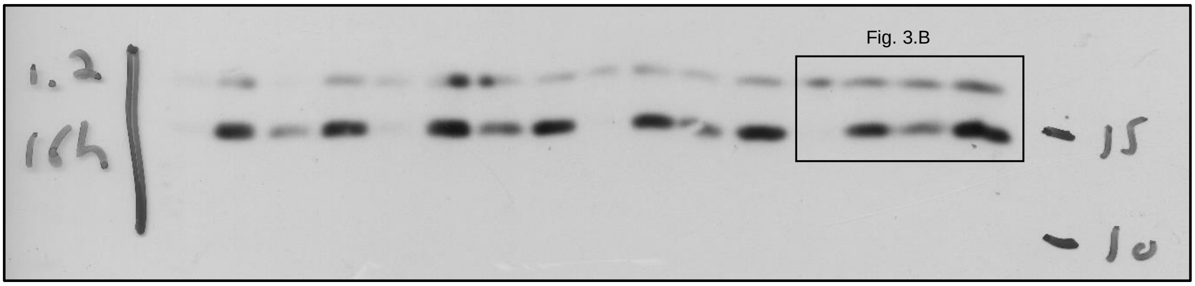

Figure 7A: original blots in different exposures

Suppl. Fig. 9

(1) GAPDH

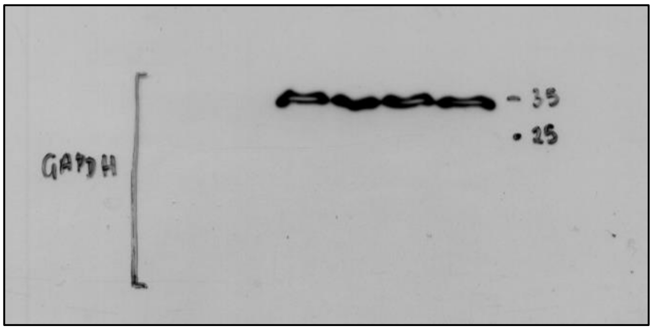

(2) GAPDH

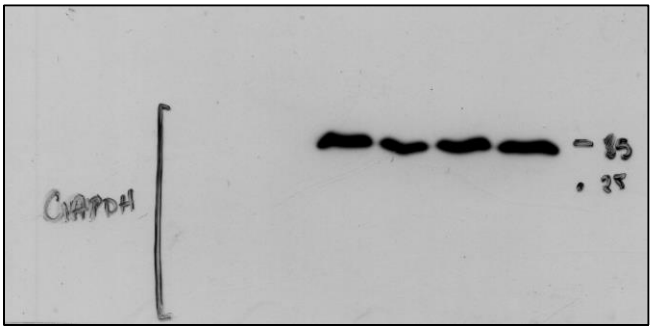

(3) RFP

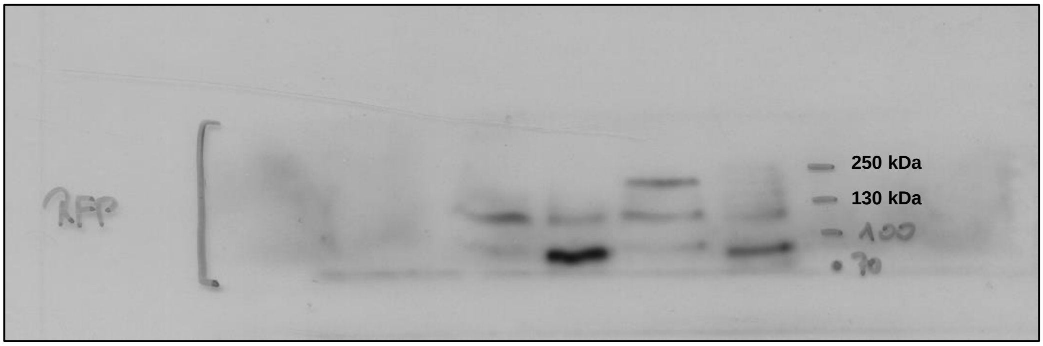

(5) RFP

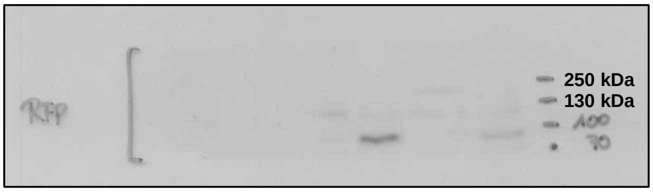

(6) ATG5

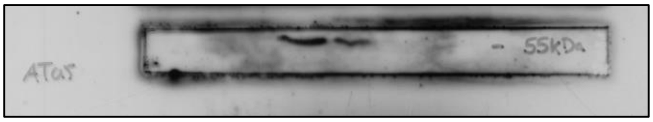

(4) RFP, ATG5

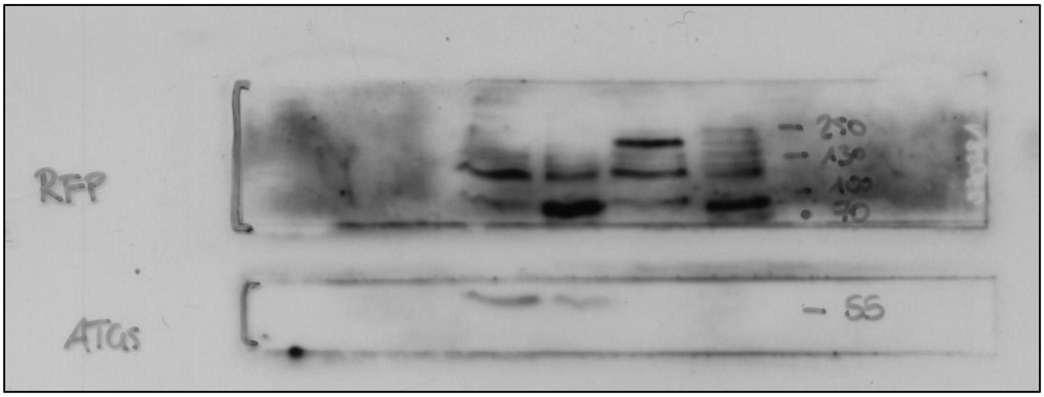

Figure 7C: original blots in different exposures

Marked samples are part of the present study. Lysates were loaded onto two different gels (#1 and #2).
#1 and #2 indicates which proteins were detected from the respective gel.

(1) GAPDH (#1), GAPDH (#2)

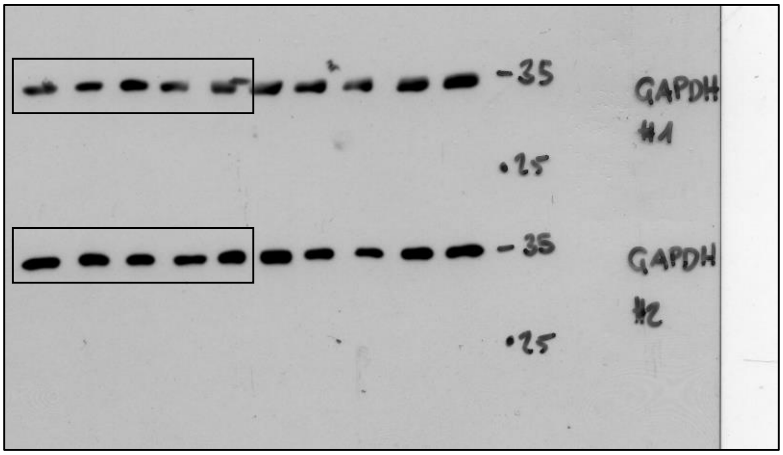

(2) Mitofusin-2 (#1), GAPDH (#1), GAPDH (#2)

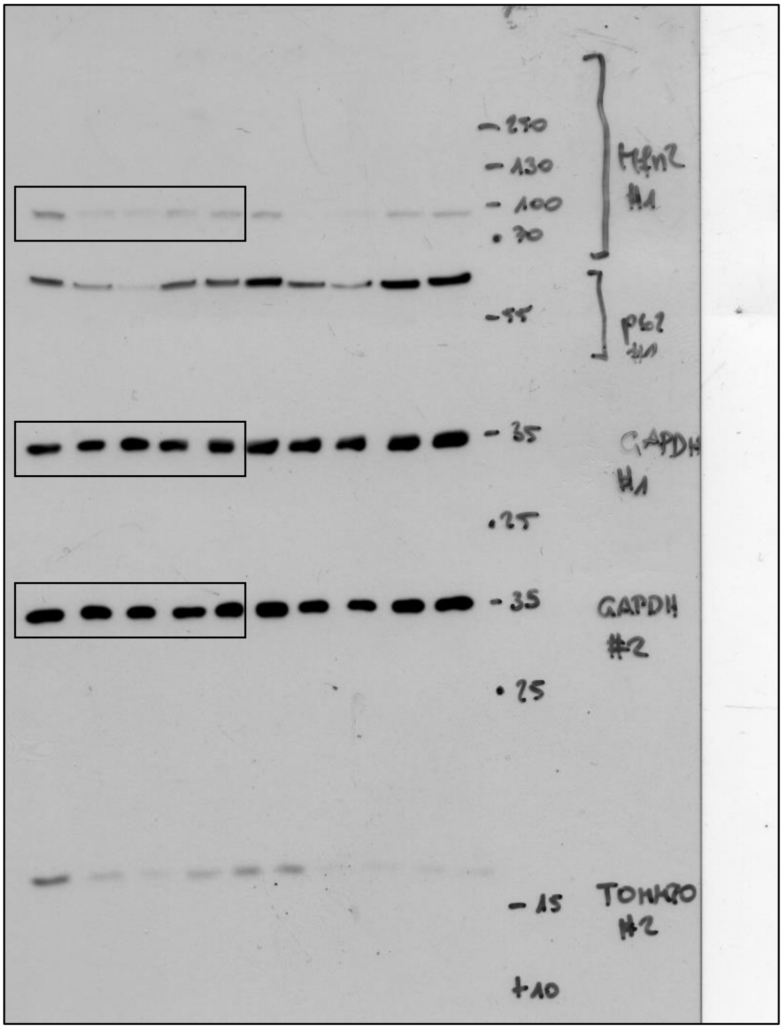

Marked samples are part of the present study. Lysates were loaded onto two different gels (#1 and #2).
#1 and #2 indicates which proteins were detected from the respective gel.

(3) Mitofusin-2 (#1), GAPDH (#1), GAPDH (#2)

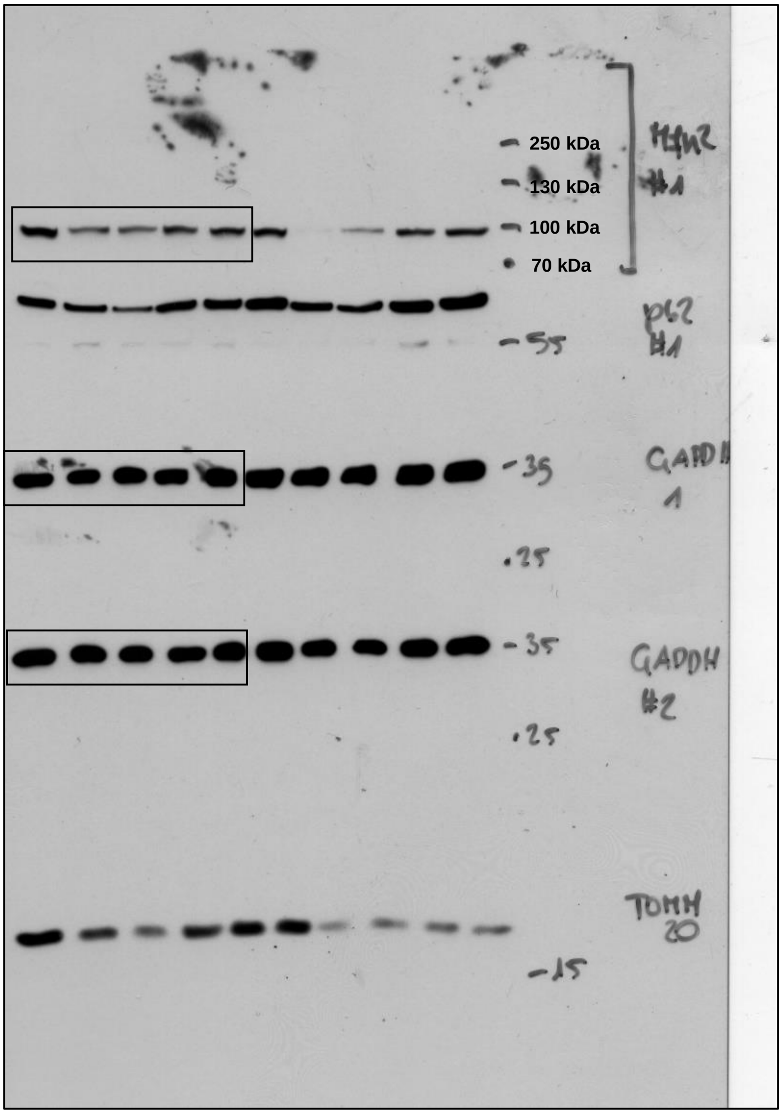

Figure 7C: original blots in different exposures

Marked samples are part of the present study. Lysates were loaded onto two different gels (#1 and #2).
#1 and #2 indicates which proteins were detected from the respective gel.

(4) COX-IV (#1)

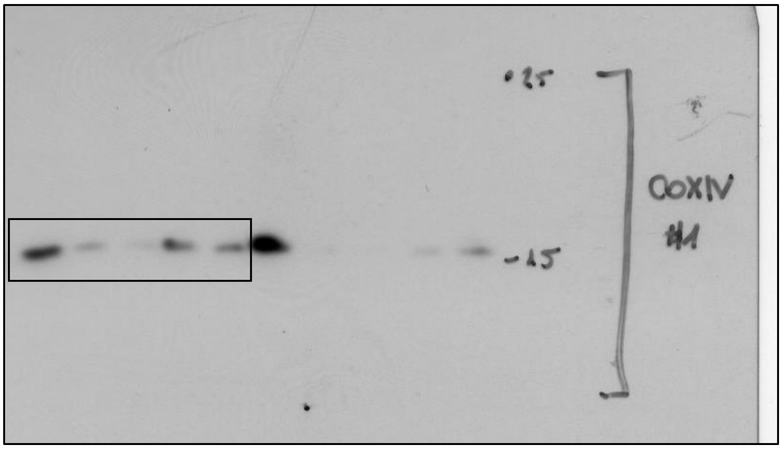

(5) LC3B (#2)

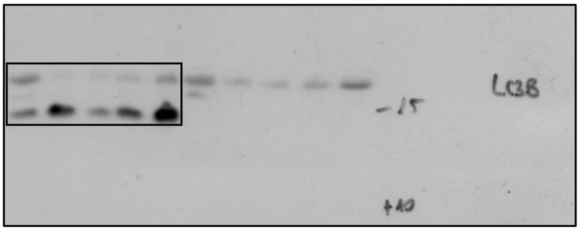

(6) LC3B (#2)

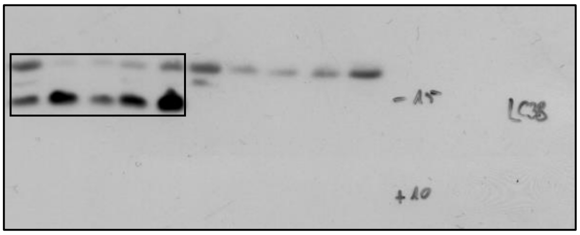

(7) COX-IV (#1), RFP (#2), ATG5 (#2)

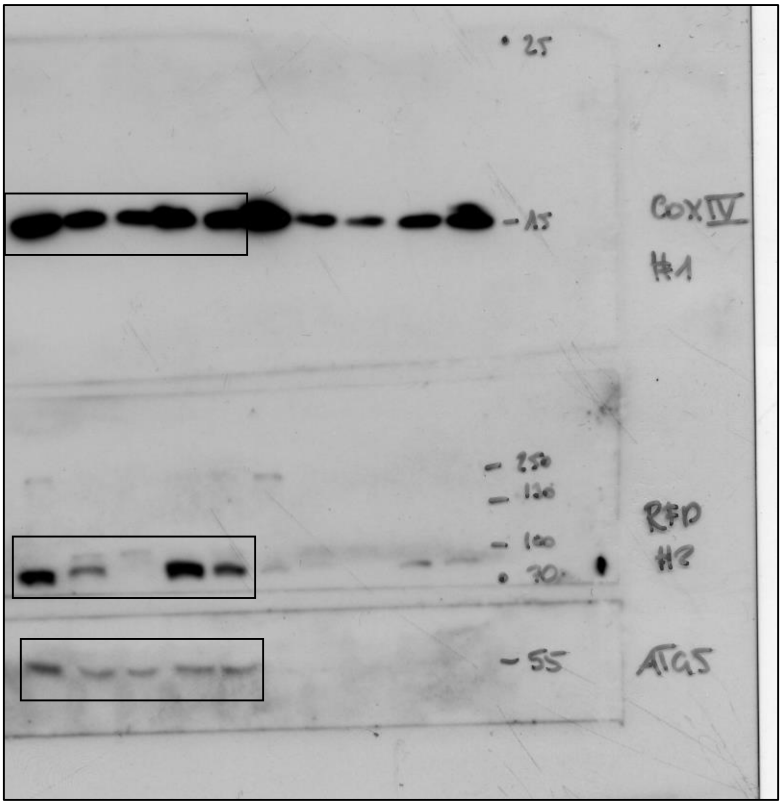

<u>Suppl. Fig. 1: original blots in different exposures</u>

Marked samples are part of the present study.

(1) Mitofusin-2

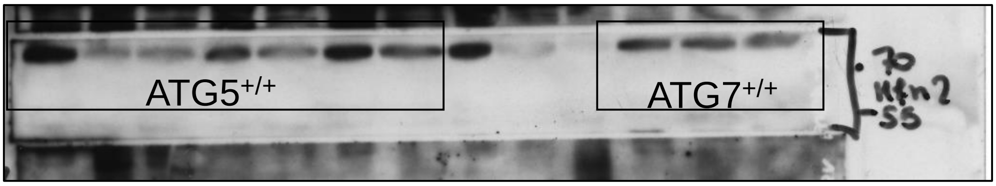

(2) mCherry-Parkin

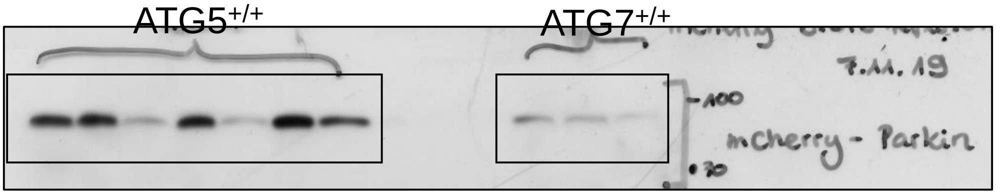

(3) COXIV

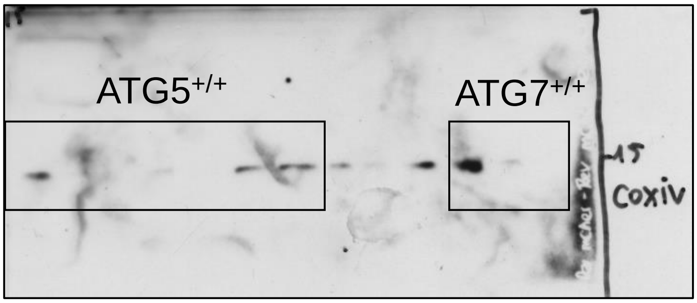

(4) LC3B

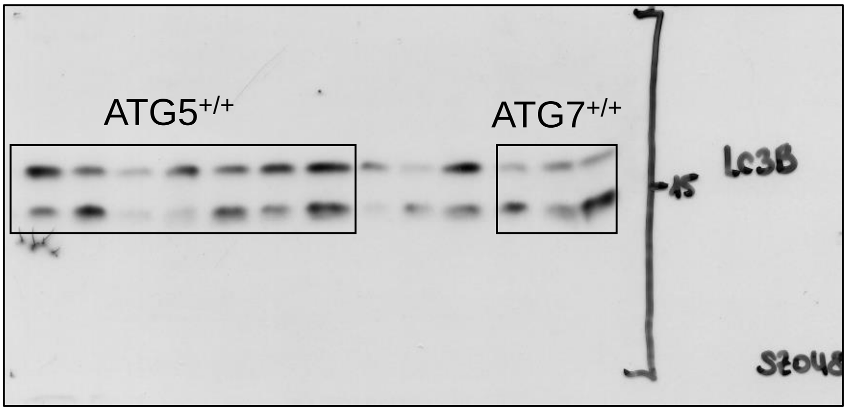

(5) Vinculin

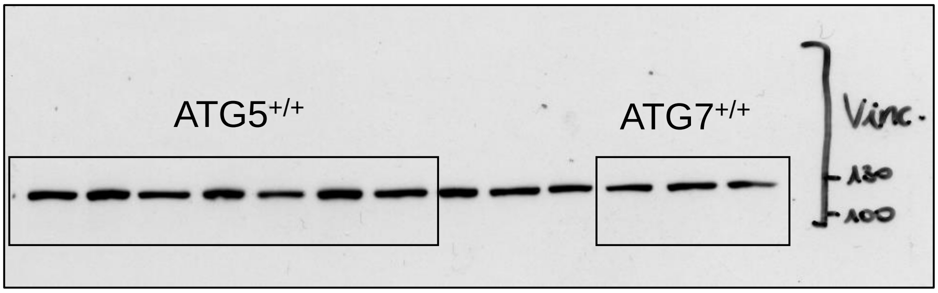

Suppl. Fig. 2: original blots in different exposures

(1) FK2 (total ubiquitin)

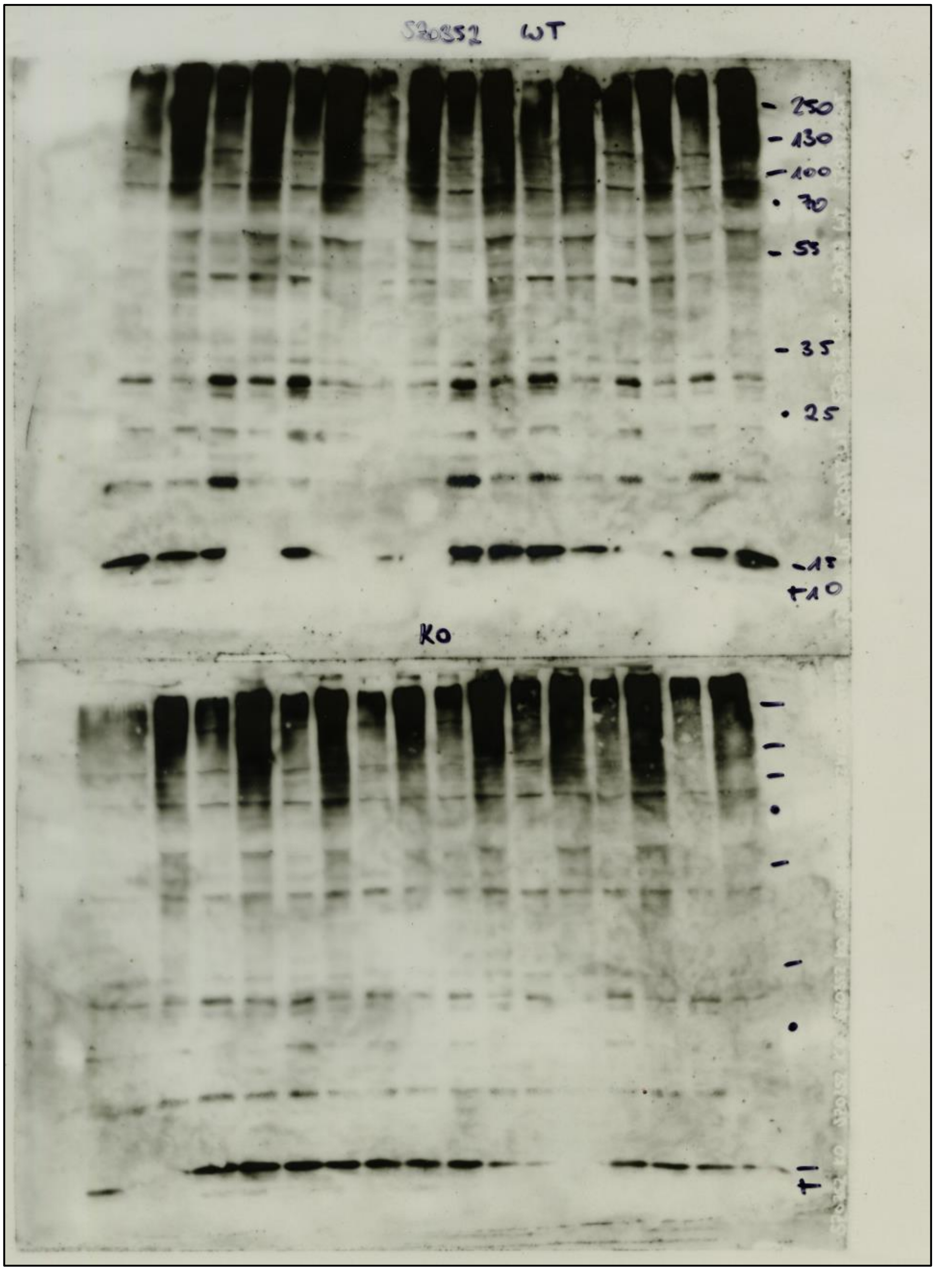

(2) GAPDH

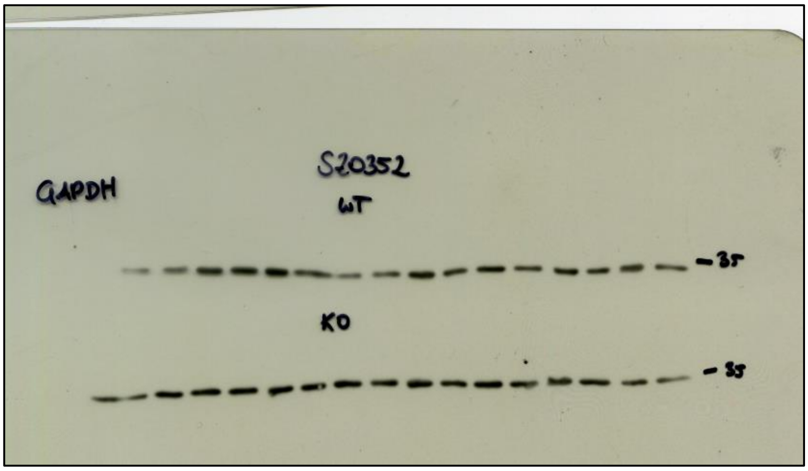

Supplement: Supplementary file 1 — Supplementary Information. [file 41598_2019_56990_MOESM1_ESM.pdf]
